# Optimized Detection of Left Ventricular Hyperpolarized [1‐^13^C]Pyruvate Signal in Human Cardiac Metabolic Imaging

**DOI:** 10.1002/mrm.70332

**Published:** 2026-03-06

**Authors:** Fatemeh Khashami, Ivan E. Dimitrov, Maximilian Fuetterer, Stefan Glöggler, Bei Zhang, Egzona Tan, Sebastian Kozerke, Anke Henning, Tarique Hussain, Craig R. Malloy, Nisha Unni, Vlad G. Zaha, CTOX Trial Investigator(s), Sheeba Cantanelli, Isaac Chan, Suzanne Cole, Suzanne Conzen, Glenda Delgado, Deborah Farr, Joshua Gruber, Barbara Haley, Dawn Klemow, Jenny Li, Heather McArthur, Alka Mallik, Poorni Manohar, Ina Patel, Namrata Peswani, Sangeetha Reddy, Navid Sadeghi, Samira Syed

**Affiliations:** ^1^ Department of Internal Medicine University of Texas Southwestern Medical Center Dallas Texas USA; ^2^ Advanced Imaging Research Center, University of Texas Southwestern Medical Center Dallas Texas USA; ^3^ Philips Cambridge Massachusetts USA; ^4^ Institute for Biomedical Engineering, University and ETH Zurich Zurich Switzerland; ^5^ Department of Pediatrics University of Texas Southwestern Medical Center Dallas Texas USA; ^6^ Dallas VA Medical Center Dallas Texas USA; ^7^ Harold C. Simmons Comprehensive Cancer Center, University of Texas Southwestern Medical Center Dallas Texas USA; ^8^ Parkland Health Dallas Texas USA

**Keywords:** carbon‐13 magnetic resonance spectroscopy, gated blood pool imaging, left ventricular blood pool detection, mathematical model

## Abstract

**Purpose:**

This study aimed to improve the analysis of the left ventricular (LV) blood pool hyperpolarized [1‐^13^C]pyruvate signal for a more reliable quantification of downstream metabolic outcome.

**Methods:**

Hyperpolarized [1‐^13^C]pyruvate magnetic resonance spectroscopic images were acquired dynamically at 3 Tesla in 106 participants (NCT03685175). An exponential model was fitted to the distribution of summed LV blood pool [1‐^13^C]pyruvate signal intensities from all dynamic frames to extract a characteristic numerical fitting value. A threshold formula was introduced to detect a suitable region of interest in the blood pool. A correlation‐based analysis between the LV blood pool [1‐^13^C]pyruvate signal and the metabolic outcome, i.e., combined myocardial [1‐^13^C]lactate and [^13^C]bicarbonate signals, was performed to determine an optimal threshold and was compared to conventional peak‐intensity and percentile‐based approaches.

**Results:**

The exponential numerical fitting value (0.0105 ± 0.0036) of [1‐^13^C]pyruvate signal intensity distribution was normalized to the area under the curve of the average signal‐to‐noise ratio. A threshold range of 13%–25% was identified where the resulting values representative for the LV blood pool [1‐^13^C]pyruvate signal reached an optimal correlation with the metabolic outcome. The proposed model showed a more stable correlation between LV blood pool [1‐^13^C]pyruvate signal and the metabolic outcome compared to conventional peak‐intensity and percentile‐based approaches.

**Conclusions:**

A mathematical model using exponential fitting and thresholding of the intensity distribution of [1‐^13^C]pyruvate signal in the LV blood pool provides a strong correlation with the metabolic outcome, allowing for a semiautomated method to robustly assess the left ventricular pyruvate signal in dynamic nuclear polarization studies.

## 1 Introduction

Hyperpolarized (HP) [1‐^13^C]pyruvate magnetic resonance spectroscopic imaging (MRSI) enables real‐time, noninvasive assessment of myocardial metabolism by tracking pyruvate conversion to [^13^C]bicarbonate and [1‐^13^C]lactate. Correlating pyruvate delivery with downstream bicarbonate and lactate production is key for reproducibility and for comparing metabolic activity among study participants [1, 2, 3]. This correlation, widely used across biological systems, provides a reliable measure of system performance and signal consistency [4, 5]. In dynamic imaging techniques such as positron emission tomography [6, 7, 8] and contrast‐enhanced MRI [9, 10], signals are typically derived from tracer or contrast agent within the left ventricular (LV) blood pool, often using automated segmentation and dynamic sampling [11, 12]. However, the fast *T*_1_ relaxation time (∼30–35 s at 3 T) and the variable first‐pass delivery, influenced by cardiac function, injection efficiency, and cardiac and respiratory cycles, complicate the temporal sampling [13, 14, 15, 16, 17] of the HP [1‐^13^C]pyruvate signal, requiring efficient data‐acquisition strategies [7, 9, 11, 18, 19, 20, 21].

The HP [1‐^13^C]pyruvate signal region‐of‐interest (ROI) has been estimated using manual segmentation of the LV blood pool or semi‐automated methods based on signal intensity threshold [13, 14], voxel‐wise kinetic modeling [22, 23, 24, 25], or pre‐defined vascular masks [11, 26]. These approaches are limited by low spatial resolution, partial‐volume effects [27, 28] from adjacent myocardium, respiratory motion, and hardware‐related factors, such as B_0_ and B_1_^+^ inhomogeneity [29, 30, 31, 32, 33], which reduces the LV pyruvate signal accuracy [19, 20, 34, 35]. Motion correction, post‐processing denoising, signal filtering, and interpolation [16, 36, 37] aim to improve signal resolution and minimize noise artifacts [23, 24, 36, 38]. While image acquisition optimization and image processing techniques enhance signal‐to‐noise ratio (SNR), the variation in HP^13^C‐CMR signal within the LV blood pool is also a potentially contributing factor, currently insufficiently characterized.

To address the HP^13^C‐CMR pyruvate delivery definition systematically, this study sought to use mathematical modeling of the distribution of pyruvate signal intensities in the LV blood pool ROI and to identify an optimal threshold to exclude low SNR voxels and prioritize high‐intensity signals that best represent pyruvate delivery. This mathematical model was then compared with a conventional peak‐intensity‐based and a percentile‐based model, assessing the correlations with the LV pyruvate signal across different thresholding percentages.

## 2 Methods

### 2.1 Study Design

A total of 106 HP [1‐^13^C]pyruvate MRSI studies were analyzed, completed in 53 participants, 59 before and 47 after different standard of care therapies for breast cancer (ClinicalTrials.gov ID: NCT03685175) [39, 40, 41, 42]. Of these, 16 studies were repeated to address technical limitations (Table S2). All study procedures were approved by the Institutional Review Board at UT Southwestern Medical Center [21, 42, 43]. Written informed consent was obtained from all participants. The HP [1‐^13^C]pyruvate samples were prepared using the SPINlab polarizer (GE Healthcare) over an average duration of 3.58 ± 0.56 h [42], achieving a mean polarization level of 36.1% ± 9.2%, measured immediately before sample transfer to the scanner using a Spinsolve NMR (Magritek, Germany). Samples were administered to study participants within 60 ± 17.7 s from dissolution.

### 2.2 Data Acquisition

#### 2.2.1 ^1^H‐CMR Acquisition

**FIGURE 1:**
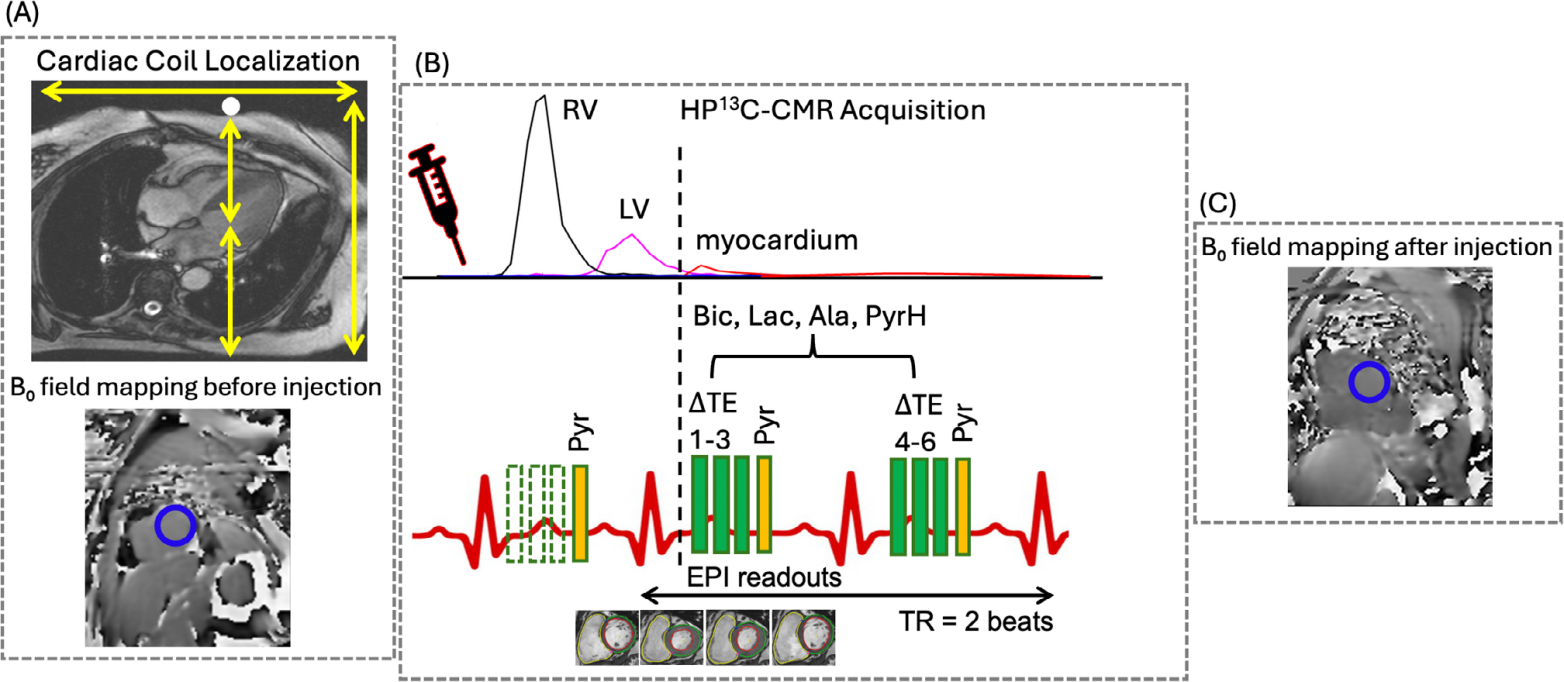
Experimental schematic showing cardiac coil localization, field mapping, and HP^13^C‐CMR acquisition protocol. (A) Top: localization of the ^13^C cardiac coil on a ^1^H cardiac long‐axis cine image; the yellow arrows indicate the coil coverage over the heart in the left–right and superior–inferior directions, and the white circle marks the 5 M [1‐^13^C]urea phantom at the center of the top coil. Bottom: corresponding pre‐injection B_0_ field map, with the blue circle highlighting the LV region on the B_0_ map. (B) Schematic of the HP^13^C acquisition protocol. Following injection of HP [1‐^13^C]pyruvate, only metabolic signals from Pyr were excited for 30 heartbeats to detect baseline metabolite noise, and then together with downstream metabolites Lac, Ala, Bic, PyrH over the last 14 heartbeats (starting at dashed line). Signal was acquired using EPI readouts, gated over two heartbeats. (C) Post‐injection B_0_ field map acquired. Ala, alanine; B_0_, static magnetic field; Bic, bicarbonate; EPI, echo‐planar imaging; Lac, lactate; LV, left ventricular; Pyr, pyruvate; PyrH, pyruvate hydrate; RV, right ventricular; TE, echo time; TR, repetition time.

Studies were conducted on a 3 T scanner (Achieva, Philips) using a proton cardiac coil (Philips, NL) and ^13^C transmit‐receive Helmholtz loop‐pair coil (PulseTeq, UK) [21, 43]. Radiofrequency (RF) calibration was performed, using a thermal torso phantom containing 1 M [1‐^13^C]pyruvate, 1 M [1‐^13^C]acetate, and 1 M [1‐^13^C]urea, simulating different human torso anterior–posterior sizes for an optimal 90‐degree reference scale (Figure S1). A thermal 5 M [1‐^13^C]urea phantom, with the urea resonance centered at 4100 Hz, was placed at the center of the top ^13^C cardiac coil used as a quantification marker (Figure 1A and S1), to verify the coil efficiency prior to HP^13^C‐CMR/MRS acquisitions. Figure 1A top shows the ^13^C cardiac coil positioned over the chest on a ^1^H long‐axis cine image; the yellow arrows indicate the coil coverage over the heart in the left–right and superior–inferior directions toward the center of the heart. First‐order shimming and B_0_ mapping before pyruvate injection were used to correct off‐resonance effects [32, 44, 45, 46] (Figure 1A bottom), and the resulting first‐order shim parameters were applied prior to the HP^13^C‐CMR/MRS acquisitions. The offset frequency 4320 Hz was selected to center the pyruvate signal for detecting the [^13^C] bicarbonate signal. ^1^H‐CMR short‐axis cine image was acquired for anatomical reference and cardiac region segmentation using a 350 × 350 mm^2^ field of view (FOV), 432 × 432 matrix size, 30 mm slice thickness to match a similar ^13^C acquisition, 2.76 ms repetition time (TR), 1.38 ms echo time (TE), and 20° flip angle [47]. Data were analyzed using CVi42 (Circle Cardiovascular Imaging Inc., Canada) for ^1^H‐CMR, and Mnova (Mestrelab Research, Spain) for MR spectra.

#### 2.2.2 HP^13^C‐CMR Acquisition

A spectral‐spatial (SP‐SP) excitation pulse sequence combined with single‐shot echo‐planar imaging (EPI‐IDEAL) readout was used for HP^13^C‐CMR [21, 43, 48, 49] (Figure 1B). Briefly, six echo‐shifted images were acquired per frame over two consecutive heartbeats, with 1.15 ms ΔTE. The excitation employed a 1‐2‐1 binomial spectral‐spatial RF pulse (BW_SP‐SP_ = 20 ppm), yielding a 30° flip angle for bicarbonate and lactate, without excitation of pyruvate [28, 50, 51, 52]. Pyruvate images were acquired in an interleaved fashion using a 5° flip angle sinc pulse. Also, a Half‐scan of k‐space was applied to record the acquisition 44 × 43 matrix size and decreased the readout duration to about 30 ms. Imaging was performed using a single‐shot EPI readout (220 × 220 mm^2^ FOV, 5 mm in‐plane resolution, 30 mm slice thickness, 0.75 partial Fourier factor), with BW_EPI_ = 0.89 ppm = 28.8 Hz, 11.6 ms TE, and TR of 1 and 2 heartbeats for pyruvate and metabolites, respectively. These optimizations improved image quality and reduced the acquisition time. A total of 44 frames (heartbeats) were acquired using the low flip angle sinc pulse to capture the full HP^13^C pyruvate bolus dynamics. SP‐SP excitation was suspended for the first 30 heartbeats to capture baseline noise. SP‐SP excitation was active for the last 14 cardiac cycles to detect downstream metabolites (vertical dashed line in Figure 1B). Metabolic images were then reconstructed for every two cardiac cycles. To illustrate the temporal relationship between pyruvate and metabolite signals, all 22 reconstructed metabolic images are presented alongside the acquired 44 frames. Compared with previous work [21, 43, 53], we analyzed 44 pyruvate frames, including the baseline‐noise frames and the physiologic delay between the pyruvate and metabolite peaks, to better characterize the relationship between the LV pyruvate signal and the downstream metabolic response and to capture sufficient time for metabolic conversion and maximize SNR. A B_0_ map was acquired after the HP^13^C‐CMR acquisition data to confirm spatial consistency (Figure 1C).

### 2.3 Data Analysis

#### 2.3.1 Region of Interest Definition

**FIGURE 2:**
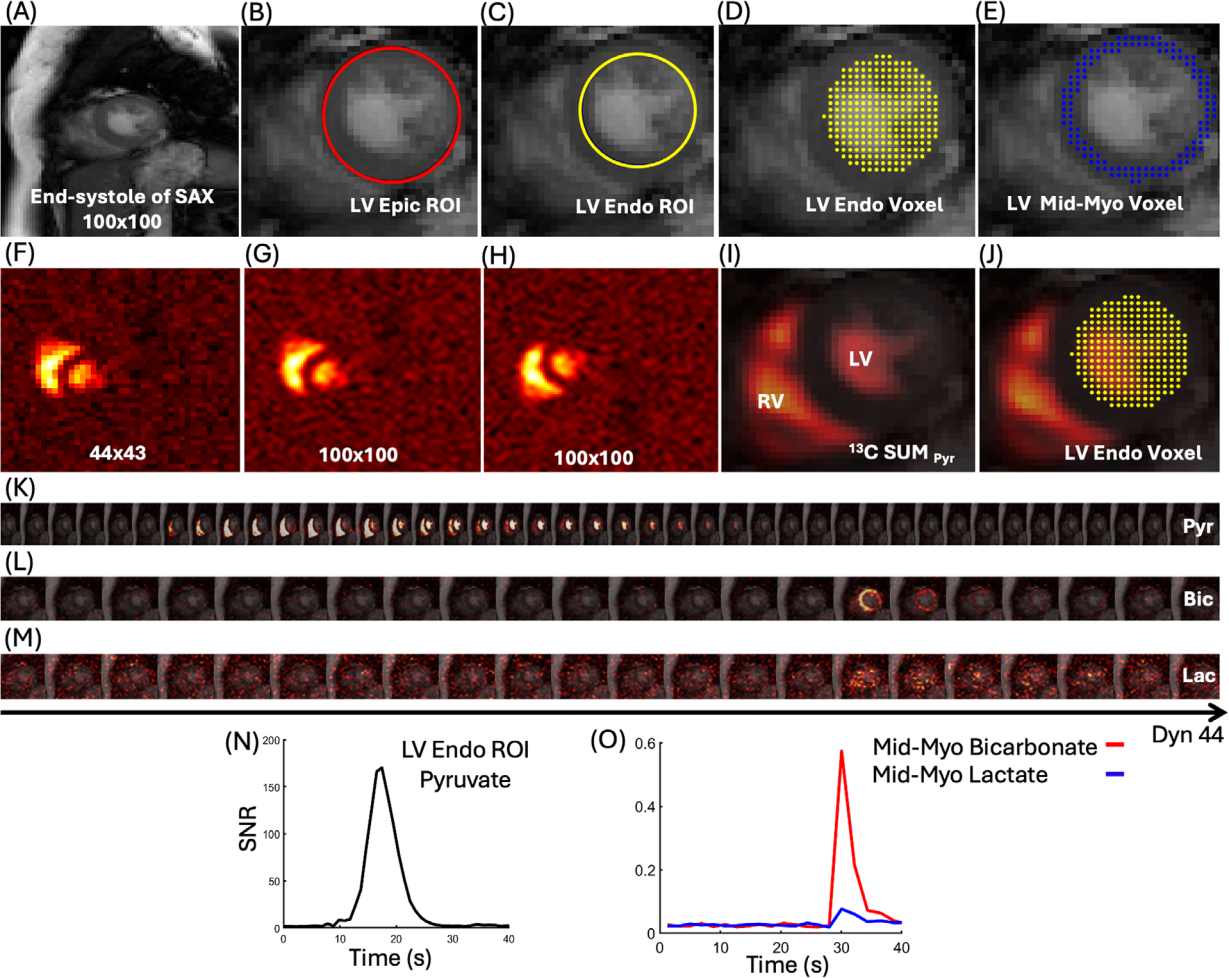
(A) Anatomical short‐axis ^1^H‐CMR image at end‐systole (100 × 100), used as anatomical reference for co‐registration. (B) Manual identification of the LV Epic ROI on the ^1^H‐CMR image. (C) Automatically detected LV Endo ROI based on the Epic ROI. (D) LV Endo ROI voxels displayed as a yellow dot mask. (E) Mid‐Myo ROI voxels are shown as a blue dot mask. (F) Reconstructed the raw data in 44 × 43. (G, H) Original 44 × 43 pyruvate image zero‐filled to 100 × 100, rotated and vertically flipped for rigid co‐registration. (I) ${\mathrm{SUM}}_{\mathrm{Pyr}}$ image co‐registered to the anatomical ^1^H‐CMR image. (J) LV Endo ROI overlaid on the ${\mathrm{SUM}}_{\mathrm{Pyr}}$ image. (K) Dynamic evolution of the pyruvate signal over 44 frames. (L, M) The dynamic evolution of bicarbonate and lactate signals is shown across 22 frames. (N) Pyruvate time‐intensity curve. (O) Bicarbonate and lactate time‐intensity curves. Bic, bicarbonate; Dyn, dynamic; Endo, endocardial; Epic, epicardial; Lac, lactate; LV, left ventricular; Mid‐Myo, mid‐myocardium; Pyr, pyruvate; RV, right ventricular; SAX, short‐axis.

^1^H‐CMR short‐axis image (originally 432 × 432 matrix size) was downsampled to a 100 × 100 matrix size to match the spatial layout of the HP^13^C pyruvate images (Figure 2A). The cine frame for metabolic data acquisition was ECG triggered at end‐systole to correspond to the end‐systolic acquisition timing of the metabolic data [2, 53, 54]. The LV epicardial (Epic) ROI was manually outlined on the ^1^H‐CMR image using an elliptical drawing tool in MATLAB (Figure 2B). The LV endocardial (Endo) ROI was then defined algorithmically based on the Epic contour by reducing the radius [28, 55] (Figure 2C,D). The myocardium was then segmented by subtracting the LV Endo from the LV Epic ROI. To exclude partial volume voxels near the Epic and Endo contours [27, 28, 54] (Figure 2E), myocardial boundaries were reduced by 10%, and this region was defined as the mid‐myocardium (Mid‐Myo) ROI [42, 56, 57].

#### 2.3.2 Image Reconstruction

HP^13^C‐CMR raw data were reconstructed into a 44 × 43 matrix size in MRecon (GyroTools LLC) using a customized conjugate‐gradient reconstruction algorithm [21, 43, 51]. The ^13^C images were interpolated and zero‐filled to a 100 × 100 matrix size. For each dynamic frame, images were corrected for flip angle by dividing by sin(θ), where θ is the excitation flip angle (5° for pyruvate and 30° for bicarbonate and lactate) [17]. All ^13^C data were acquired from a short‐axis slice with prospective ECG triggering at end‐systole (Figure 2A) [28, 50, 58]. The reconstructed ^13^C images were rigidly co‐registered to match the geometry of the corresponding ^1^H‐CMR anatomical reference, using horizontal or vertical flip in addition to 90‐degree rotation, as needed, with MATLAB image‐registration tools. Summed pyruvate (${\mathrm{SUM}}_{\mathrm{Pyr}}$) image was derived from all 44 pyruvate frames [48, 59], and is shown together with the LV Endo voxel mask in Figure 2F–J. The same reconstruction and alignment procedure was applied to the bicarbonate and lactate metabolic images to ensure spatial consistency, and Figure 2K–M presents the full 44‐frame dynamic series for pyruvate, 22‐frame for bicarbonate, and lactate.

#### 2.3.3 Quantitative Analysis

HP^13^C‐CMR images were quantified using the defined ROIs. For pyruvate, voxel‐wise SNR values were calculated within the LV Endo ROI across all 44 dynamic frames to generate an average pyruvate SNR time curve, and the average area under the curve (AUC) was then calculated [48, 55, 59]. A similar approach was applied to the bicarbonate and lactate signals using the Mid‐Myo ROI [21, 42, 43, 54]. Figure 2N,O displays the time courses for both pyruvate and metabolites outcomes [21, 43].

### 2.4 Mathematical Modeling

#### 2.4.1 Exponential Modeling of LV Pyruvate Signal Distribution

**FIGURE 3:**
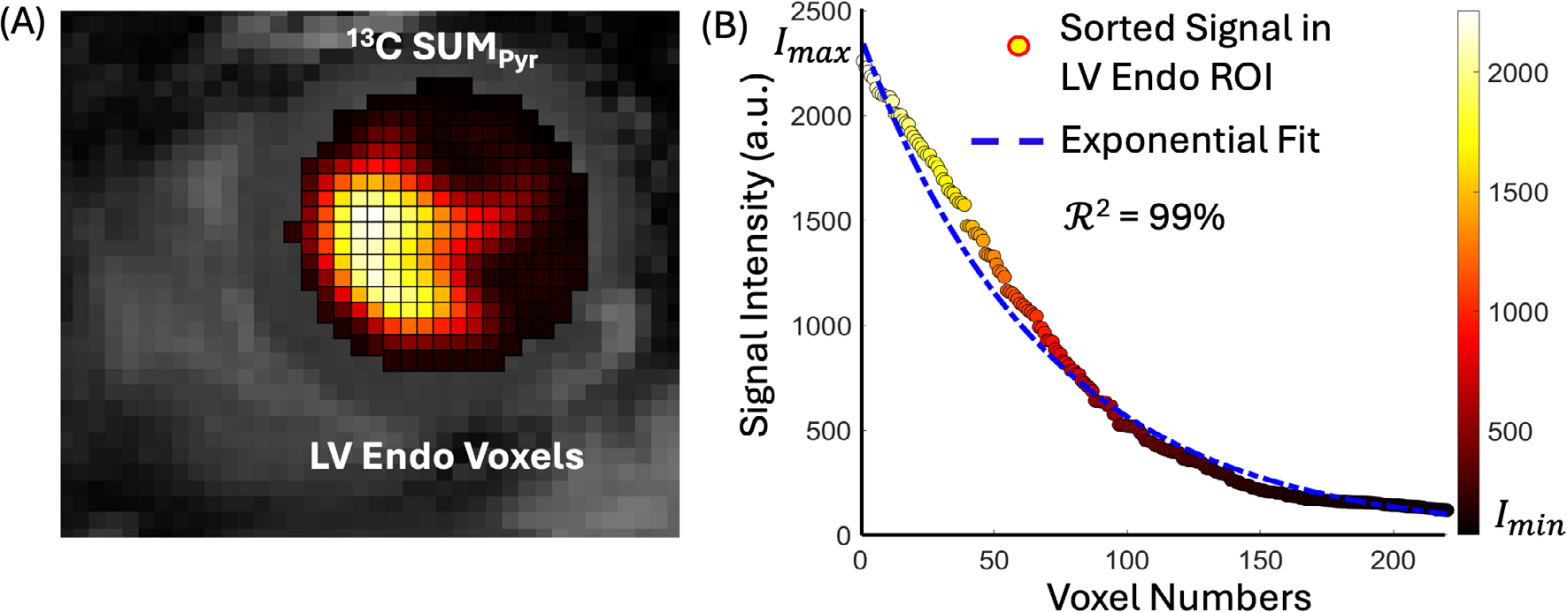
(A) Overlay of ^13^C ${\mathrm{SUM}}_{\mathrm{Pyr}}$ image within the LV Endo ROI on the anatomical ^1^H‐CMR image. (B) Sorted pyruvate voxel intensities plotted against signal voxel numbers, with the distribution fitted to an exponential model. The blue dashed line represents an exponential fit $\left(R^{2}=99\%\right).I_{\max}$ and $I_{\min}$ are marked on the plot as maximum and minimum intensity, respectively. a.u., arbitrary units; Endo, endocardial; LV, left ventricular; Pyr, pyruvate; ${\mathrm{SUM}}_{\mathrm{Pyr}}$, summed pyruvate.

Understanding the distribution of the pyruvate signal within the ROI of the LV blood pool is crucial for optimizing the LV blood pool pyruvate signal. To evaluate LV pyruvate signal distribution, the LV Endo ROI was overlaid onto the ${\mathrm{SUM}}_{\mathrm{Pyr}}$ image (Figure 3A), and voxel intensities within the ROI were sorted in descending order as

(1) $$I_{\max}=I_{1}\geq I_{2}\geq \cdots \geq I_{N}=I_{\min},$$

where N is the total number of voxels in the selected area, $I_{\max}$ is the maximum single‐voxel SNR within the ROI $I_{\max}=I_{1}$, and $I_{\min}$ is the lowest signal intensity voxel within the ROI $I_{\min}=I_{N}$ (Figure 3B). We showed that the sorted signal intensity of all voxels in the LV Endo ROI follows an exponential distribution. To be more precise, the exponential distribution of the j‐th signal intensity ($I_{j}$) within the LV Endo ROI [60, 61, 62] can be modeled as

(2) $$I_{j}=I_{\max}e^{-B\left(j-1\right)},j=1,\ldots ,N$$

where B is the numerical fitting value of the LV pyruvate signal that characterizes the spatial distribution of the pyruvate signal across voxels. The exponential fitting is characterized by the numerical fitting value, which can be determined from the experimental data (Figure 3B). The agreement between the exponential fit and the sorted intensities was estimated using the coefficient of determination, $R^{2}=$ 0.97 ± 0.03. This exponential pattern was observed consistently in all 106 scans (Figure S2). The fitted parameter serves as a compact, single‐parameter metric of LV pyruvate distribution that can be compared across subjects, visits, and injections. A comparison between the raw data, Equation (2), and the fitted parameter for representative cases is presented in Figure S3.

#### 2.4.2 Thresholding Approach for LV Blood Pool ROI Selection

HP [1‐^13^C]pyruvate in the LV blood pool includes regions with high tracer delivery activity and low SNR voxels near the ROI borders (LV Endo ROI, Figure 2D) [27, 28]. The low‐SNR voxels affect the reliability of the analysis. To systematically exclude the low SNR voxels, a thresholding formula is proposed based on the LV Endo ROI ${\mathrm{SUM}}_{\mathrm{Pyr}}$ image, expressed as

(3) $${\mathrm{SUM}}_{\mathrm{Pyr}}>\alpha I_{\max}+I_{\min},$$

where α is the percentage of the maximum intensity $I_{\max}$. This threshold captures high‐intensity voxels and includes a baseline adjustment ($I_{\min}$) to account for background noise. Since some studies have fewer high‐intensity voxels within the ROI, $\alpha_{\max}$ is calculated individually for each study using the condition $\max\left({\mathrm{SUM}}_{\mathrm{Pyr}}\right)=I_{\max}$ in Equation (3), given by

(4) $$\alpha_{\max}=\frac{I_{\max}-I_{\min}}{I_{\max}}\times 100.$$

**FIGURE 4:**
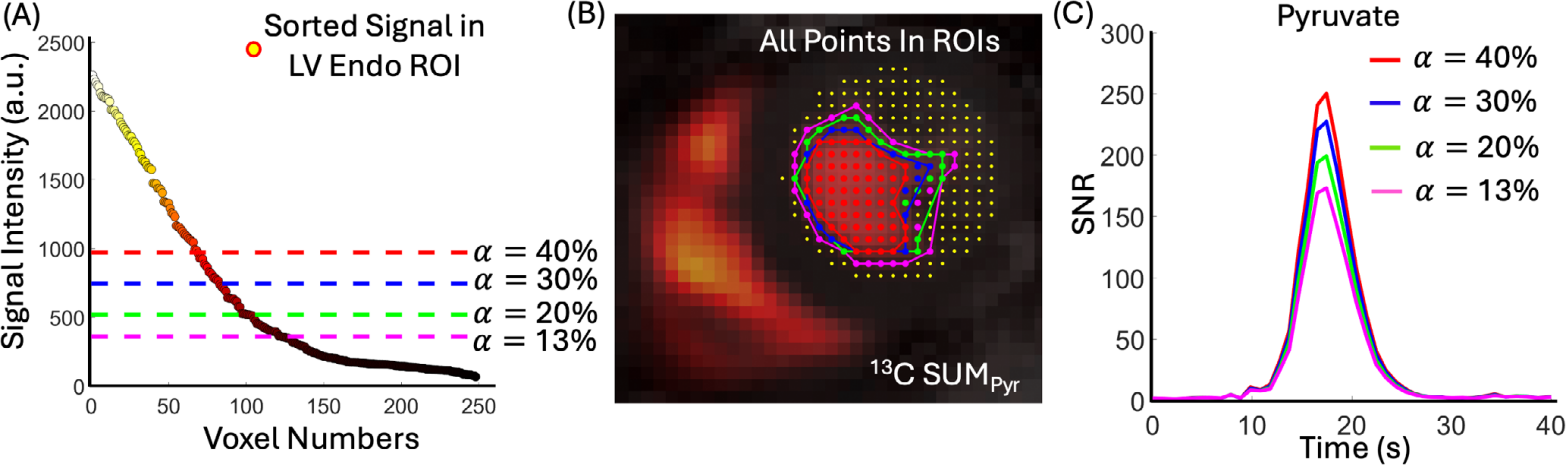
Visualization of the LV blood pool ROI detection and thresholding approach in the LV Endo ROI. (A) Dashed lines indicate different α thresholds. (B) Illustration of ROI boundaries and all included voxels. (C) Time‐intensity curves for various α thresholds. a.u., arbitrary units; Endo, endocardial; SNR, signal‐to‐noise ratio.

To account for variable SNR across studies, the minimum value $\alpha_{\max}$ across all studies was used as the upper limit. The minimum $\alpha_{\max}$ is below 64%, indicating that at least one study retained only one high‐intensity voxel within the ROI at this threshold, setting the threshold range to 1%≤α≤63%. The selected threshold ROI% is overlaid on each pyruvate frame, and time‐intensity curves were generated by averaging the voxel‐wise SNR within the ROI from each frame. The area under this average curve is then computed and denoted as Pyr(α(%)) (Figures 4 and S4).

To illustrate the thresholding approach used to define Pyr(α(%)), Figure 4 presents an example from one representative study. Panel A shows the voxel‐wise pyruvate signal intensities from ${\mathrm{SUM}}_{\mathrm{Pyr}}$ image sorted in descending order, with dashed lines marking different α thresholds (e.g., 13%, 20%, 30%, 40%). Panel B maps all voxels in the LV Endo ROI spatially, with overlays showing the subset of voxels included at each threshold. As α increases, the ROI is restricted to the brightest central voxels in the LV blood pool. Panel C shows the resulting time‐intensity curves; for each α threshold, the voxel‐wise SNR values within the corresponding ROI were averaged at each dynamic time point, and AUC were exported for further analysis.

#### 2.4.3 Correlation‐Based Comparisons of the Proposed Models and Metabolic Outcome

To identify the optimal threshold α (proposed in Equation 3) that minimizes the influence of low SNR voxels, the proposed method designed a correlation analysis between the LV pyruvate signal model and the metabolic outcome. The metabolic outcome was defined as the combined average AUCs of lactate and bicarbonate SNR (Lac+Bic) within the Mid‐Myo ROI [21, 42, 50, 59, 63]. This metabolic outcome served as a reference for evaluating the LV pyruvate signal. A stronger correlation between the LV pyruvate signal model and the metabolic outcome was considered to provide a more accurate estimation of the pyruvate signal [3, 5, 18, 19, 44, 64]. For any pair of variables X related to the LV pyruvate signal model and Y (metabolic outcome), the correlation coefficient Corr(X,Y) is defined as [62, 63].

(5) $$\text{Corr}\left(X,Y\right)=\frac{\mathrm{Cov}\left(X,Y\right)}{{\mathrm{STD}}_{X}{\mathrm{STD}}_{Y}},$$

where Cov(X,Y) is the covariance between *X* and *Y*, quantifying how changes in one variable are related to changes in the other; ${\mathrm{STD}}_{X}$ and ${\mathrm{STD}}_{Y}$ are the standard deviations of *X* and *Y*, respectively. A correlation close 0 indicates no relationship, and a value near ±1 reflects a strong negative/positive relationship [4, 5, 65].

Building on the numerical fitting value B and the proposed threshold model for defining LV pyruvate blood pool ROIs, Pyr(α(%)), we combined these two descriptors into a single dimensionless metric. B value characterizes the pyruvate distribution across voxels and encodes the shape and spatial organization of the LV pyruvate signal, whereas Pyr(α(%)) represents the overall magnitude of delivered pyruvate within the ROI at a given α. To reduce sensitivity to global scaling factors (e.g., polarization level), we defined the normalized quantity for the LV pyruvate signal,B/Pyr(α(%)), denoted as the numerical fitting model. This ratio can be interpreted as the spatial concentration of pyruvate signal per unit of delivered LV pyruvate signal. In the subsequent analysis, B/Pyr(α(%)) metric is evaluated across the full threshold range (1% ≤ α ≤ 63%) and used as the proposed numerical fitting model when examining its relationship with downstream metabolic outcome. By incorporating both aspects, spatial distribution, B value, and total delivery, Pyr(α(%)), the normalized metric provides a more meaningful basis for comparing studies and thresholds when examining the relationship between LV pyruvate signal model and Lac+Bic.

To enable a fair comparison, the proposed numerical fitting model was evaluated alongside two models, peak‐intensity‐based and percentile‐based models. As described in Equation (2), the LV pyruvate distribution is characterized by two parameters: $I_{\max}$ a global scale factor that sets the overall signal magnitude (peak intensity), and the B value, which controls the signal distribution across voxels. For consistency, the peak‐intensity‐based model, the maximum voxel intensity within the same ROI,$\;I_{\max}$, was normalized by Pyr(α(%)), giving $I_{\max}/$Pyr(α(%)). Using the same Pyr(α(%)) normalization for both B value and $I_{\max}$ puts all LV pyruvate signal models on the same scale. Because Pyr(α(%)) is an average AUC measure and $I_{\max}$ is a single‐point value, $I_{\max}/$Pyr(α(%)) can exceed 1, especially when the signal is highly concentrated in a few voxels.

In the percentile‐based model, Pyr(T(%)) was computed by selecting the top T% of voxels in the LV blood pool ROI, where T% is, for example, 10%, …, up to 100% and calculating the corresponding AUC. For this model, B/Pyr(T(%)) was computed to allow direct comparison with the proposed B/Pyr(α(%)) metric. In this framework, B value is the same exponential shape descriptor of the LV pyruvate distribution for all studies analyzed here, and the only distinction between the percentile‐based model (traditional model) and proposed approaches lies in how the pyruvate delivery term is defined: Pyr(T(%)) in the percentile‐based model versus Pyr(α(%)) in the threshold‐based model from Equation (3).

## 3 Results

### 3.1 Pyruvate Signal Characteristics and Thresholding Analysis in the LV Endo and LV Blood Pool ROIs

Table 1 summarizes the voxel‐wise pyruvate signal characteristics within the LV Endo ROI across 106 HP studies. The maximum and minimum voxel signal intensities, total pyruvate signal, and voxel count within the region highlight the heterogeneity of pyruvate uptake and signal distribution across studies. Additionally, Table 1 presents the unitless B value, along with its corresponding fit quality ($R^{2}$). Further details are provided in Table S2.

**TABLE 1:** Summary of LV Endo ROI signal intensities of 106 studies.

HP^13^C‐CMR (per voxel)	Mean ± STD	Minimum	Maximum
Maximum signal intensity, $I_{\max}$	1286 ± 942	126	5577
Minimum signal intensity, $I_{\min}$	94.9 ± 63.7	38	385
Total pyruvate signal, $I_{\text{total}}$	104 541 ± 62 966	17 437	298 969
Number of voxels, N	228 ± 40	151	315
Fit quality, $R^{2}$(%)	97 ± 3	77	99.8
Numerical fitting value, B	0.0105 ± 0.0036	0.0028	0.0183

Abbreviation: STD, standard deviation.

Table 2 details the voxel counts and signal intensities within the LV blood pool ROI across all analyzed datasets at various intensity thresholds. It reports the number of voxels (N), the corresponding threshold intensity ($\alpha I_{\max}+I_{\min}$), the total pyruvate signal above the proposed threshold model (${\mathrm{SUM}}_{\mathrm{Pyr}}>\alpha I_{\max}+I_{\min}$), and the average pyruvate SNR, Pyr(α(%)). As the threshold increases, the average voxel counts decreases, and the average threshold intensity and total pyruvate signal above the threshold exhibit corresponding changes. The table demonstrates that as a higher intensity threshold is applied, fewer voxels are included in the analysis, resulting in a lower total signal above the threshold but representing regions with more intense pyruvate presence. The changes reflect a shift from capturing the broader, less intense signal distribution at lower thresholds to focusing on the LV pyruvate signal at higher thresholds.

**TABLE 2:** Summary of LV Blood Pool ROIs from 106 studies.

α(%)	N (Voxels)	$\alpha I_{\max}+I_{\min}$ (a.u.)	${\mathrm{SUM}}_{\mathrm{Pyr}}>\alpha I_{\max}+I_{\min}$ (a.u.)	Pyr(α(%)) (a.u.)
1	224 ± 40	108 ± 70	5897 ± 2820	197.9 ± 121.6
10	160 ± 38	224 ± 143	1982 ± 2319	264.2 ± 175.2
20	117 ± 29	352 ± 233	1256 ± 1499	321.1 ± 214.4
30	89 ± 24	481 ± 326	947 ± 1114	364.7 ± 243.9
40	69 ± 20	610 ± 420	785 ± 910	401.2 ± 268.2
50	52 ± 17	738 ± 513	677 ± 783	434.4 ± 289.5
60	36 ± 15	867 ± 607	591 ± 692	467.3 ± 312.1

Abbreviations: a.u., arbitrary units; Pyr, pyruvate.

### 3.2 Characterization of the B Value in the Proposed Model

**FIGURE 5:**
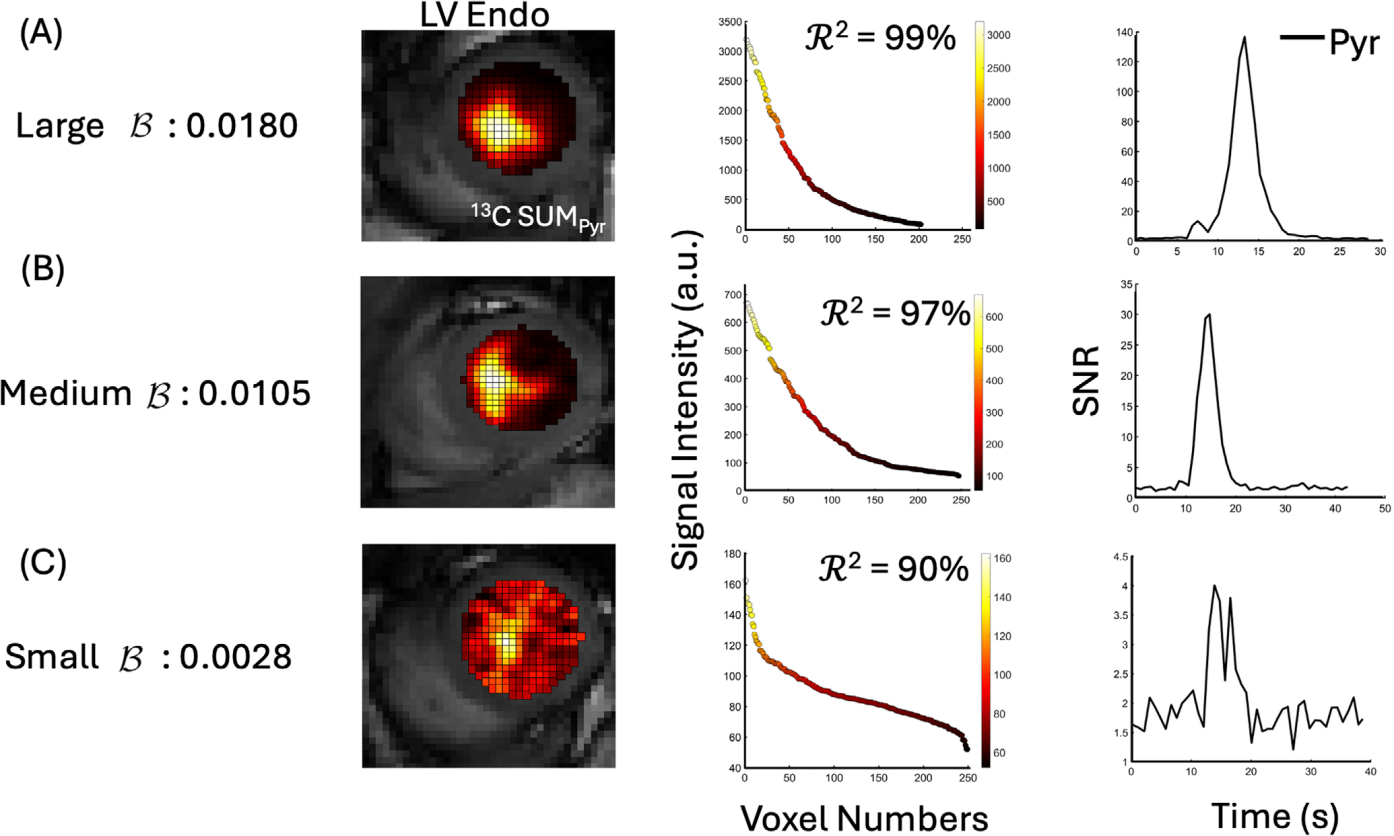
Comparative analysis of pyruvate signal distribution within the LV Endo ROI across three cases with different spatial distribution characteristics. (A) A large distribution coefficient (Large B = 0.0180) shows a strong pyruvate signal concentration in a small region. The middle panel shows a sorted voxel intensity curve with a steep slope ($R^{2}$ = 99%) of exponential behavior, and the right panel displays the corresponding time‐intensity pyruvate SNR curve. (B) Medium distribution coefficient (Medium B = 0.0105) shows a moderately concentrated signal, with a sorted voxel intensity curve with a less steep slope ($R^{2}$ = 97%) and a corresponding SNR peak. (C) Small distribution coefficient (Small B = 0.0028) illustrates a dispersed signal across the LV Endo ROI, with a sorted voxel intensity curve with a shallow slope ($R^{2}$ = 90%) and lower SNR over time. a.u., arbitrary units; Endo, endocardial; LV, left ventricular; SNR, signal‐to‐noise ratio.

The B value characterizes the spatial distribution of the pyruvate signal within the LV Endo ROI and varies across studies depending on HP signal quality. To visualize how B value relates to pyruvate signal characteristics, Figure 5 presents three representative examples of studies with small, medium, and large B values. These cases were selected to illustrate the prototypical signal distribution patterns associated with each B range in our cohort.

For large B, the pyruvate peak intensity is confined to a small region near the center of the LV blood pool. The signal color map (Figure 5A, left) highlights this concentrated signal, and the voxel intensity profile (Figure 5A, middle) curve has a steep slope. The corresponding time‐intensity curve (Figure 5A, right) shows high SNR, with a sharp, well‐defined peak and minimal baseline noise. For medium B, the spatial distribution is slightly more heterogeneous than in the large B case (Figure 5B, left and middle). The pyruvate time‐intensity curve (Figure 5B, right) still shows a dominant peak but with reduced SNR and a broader peak shape. For small B, the signal is distributed over a wide area in the LV Endo ROI. The heatmap and intensity color map (Figure 5C, left and middle) show a diffuse pattern, with only a small cluster of high‐intensity (yellow) voxels and the remaining voxels predominantly in the red–orange range; accordingly, the voxel intensity profile curve has a shallow slope. The pyruvate time‐intensity curve (Figure 5C, right) exhibits lower SNR, with substantial baseline noise, yet still shows a bolus‐shaped rise above the baseline noise in this example.

These illustrative examples highlight the qualitative differences in pyruvate signal morphology that the B value captures, ranging from a focused, high‐SNR voxel signal (large B) to broader, lower‐SNR distributions (small B). Quantitative comparisons of voxel count retained at a selected threshold, as determined by the proposed thresholding model, are presented in the next section to further illustrate how the B value shapes ROI definition.

### 3.3 Optimization of α Threshold and Comparison of LV Pyruvate Signal Models

The LV blood‐pool ROI includes both high‐SNR and lower‐SNR boundary voxels. As a result, the choice of the threshold parameter α can substantially alter the derived LV pyruvate signal model. We therefore optimized α and compared the proposed numerical fitting model to a peak‐intensity‐based model using population‐level correlations with Lac+Bic. The threshold‐dependent distributions of both metrics across ROI = 1%–63% and 106 studies are visualized in Figure S5, which provides an overview of inter‐study variability and the progressive stabilization of the metrics as α increases.

**FIGURE 6:**
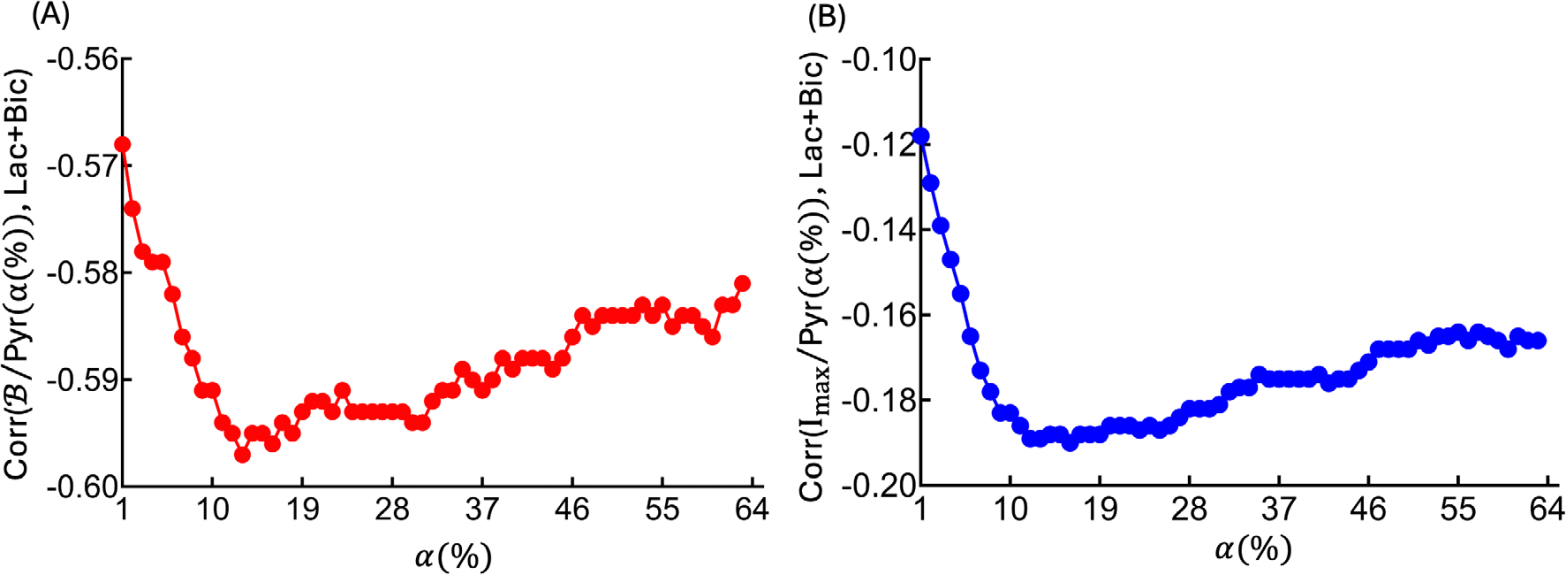
Population‐level correlation between the LV pyruvate signal model and the metabolic outcome, Lac+Bic, across thresholding percentages 1% to 63% for 106 HP studies. (A) Correlation between B/Pyr(α(%)) and Lac+Bic, numerical fitting model, (B) Correlation between $I_{\max}/$Pyr(α(%)) and Lac+Bic, peak‐intensity‐based model. Bic, bicarbonate; Lac, lactate; Pyr, pyruvate.

The analysis of the population‐level correlation between the LV pyruvate signal model and Lac+Bic as a function of α facilitates the identification of an optimal α range where the correlation is strongest. Figure 6 displays these correlations for thresholding 1%–63%. The *x*‐axis represents α(%), and the *y*‐axis shows the population‐level correlation between Lac+Bic and the two LV pyruvate signal models, the proposed numerical fitting model, and the peak‐intensity‐based model. Correlations are negative because a lower LV pyruvate signal is associated with higher Lac+Bic. The *y*‐axis range in each panel is adjusted to the dynamic range of that model's correlations so that the location of the minimum and the shape of the α‐dependence are clearly visible.

To quantitatively compare the two models over all thresholds, we performed a two‐tailed paired *t*‐test on the 63 correlation values. The proposed numerical fitting model yielded significantly stronger and less variable correlations than the peak‐intensity‐based model (−0.59 ± 0.0056 vs. −0.17 ± 0.013; *p*‐value < 0.0001), indicating that the numerical fitting model provides a stronger and more stable association with Lac+Bic across the full α range.

The α‐dependence in Figure 6 highlights the optimization range. As α increases from 1%, both curves initially decline by over 1% ≲ α ≲ 10%, indicating a noise‐dominated regime in which the selected ROI still contains a high proportion of low‐SNR voxels. In this range, noise dilutes the relationship with metabolism and drives the correlations toward weaker values. The numerical fitting model (Figure 6A) reaches its most negative correlation at α = 13%, whereas the peak‐intensity‐based model (Figure 6B) does not reach its minimum until α = 16%, indicating that the peak‐intensity‐based model is more sensitive to voxel‐level noise and requires discarding more low‐SNR voxels before stabilizing. In Figure 6A, the pattern points to a practical optimization window of α = 13%–25%, where the correlations are strongest and most stable while still retaining a reasonable number of voxels in the ROI (Tables 1 and 2). Within this window, we selected α = 20% as the suggested ROI threshold for subsequent analyses because it lies on the correlation plateau while preserving sufficient voxel counts. A Fisher *Z*‐test confirms a statistically significant difference between the correlations in the two models at this threshold (α = 20%, *p*‐value = 0.00043), with the proposed numerical fitting model showing a stronger correlation.

To illustrate the impact of the optimized threshold, at α = 1% the correlation is −0.568 for the numerical fitting model and −0.118 for the peak‐intensity‐based model, whereas at α = 20% these values change to −0.592 and −0.186, respectively. The relative correlation difference between the numerical fitting and peak‐intensity‐based models at α = 1% and α = 20% is 4% and 57%, respectively, indicating that the peak‐intensity‐based model is much more sensitive to threshold selection, while the numerical fitting model remains comparatively robust within the optimized range.

In selected example studies shown in Figure 5, at different B ranges and considering the threshold, for example, α = 20%, the LV Endo ROI retains 77 of 200 voxels for large B, 106 of 250 for medium B, and 124 of 250 for small B. These differences are consistent with the expected HP signal distribution: larger B values yield a more concentrated signal with fewer high‐SNR voxels, whereas smaller B values correspond to a broader but lower‐intensity signal spread across more voxels. This behavior reinforces the model's ability to adapt the selected ROI definition to the spatial characteristics of the underlying signal.

These optimization range and correlation analyses (Figure 6) were performed using all injections. Because several subjects contributed repeated scans across visits and injections (V1/V2, Inj1/Inj2; V1 pre‐treatment, V2 post‐treatment), measurements from the same heart may not be statistically independent [66, 67, 68], which can underestimate variability and inflate significance if ignored [67, 69]. To address this, we used three complementary approaches: (i) a primary analysis restricted to one scan per subject, (ii) linear mixed‐effects models including all 106 injections with a random intercept per subject to model within‐subject correlation [70, 71, 72, 73], and (iii) paired (V1–V2) analyses for the first injection to focus on within‐subject changes [74, 75]. Analysis details and datasets are provided in Tables S2 and S3, including linear mixed‐effects results (Figure S6) and paired (V1–V2) analyses with Bonferroni correction [76] (Figure S7). These sensitivity analyses showed that our main conclusions were robust and remained unchanged across all three approaches.

### 3.4 Comparison With the Percentile‐Based Model

**FIGURE 7:**
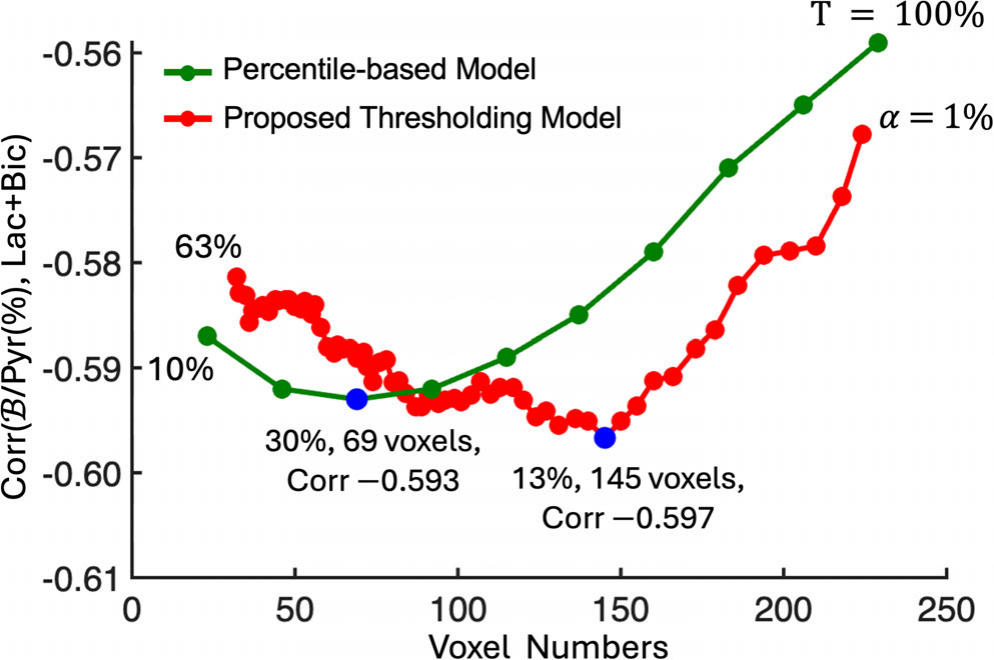
Comparison Corr(B/Pyr(%), Lac+Bic) versus the number of voxels in the LV blood‐pool ROI using the percentile‐based model (green curve) considers the top T% of highest‐intensity pyruvate voxels (where T = 10% to 100%, left to right), computed Pyr(T(%)), and the proposed thresholding model (red curve) is from α= 1% to 63% (right to left) to compute Pyr(α(%)). Blue points mark each model's most negative correlation: α = 13% at 145 voxels with Corr = −0.597, and T = 30% at 69 voxels with Corr = −0.593. Bic, bicarbonate; Corr, correlation; Lac, lactate; Pyr, pyruvate.

Figure 7 compares two approaches for defining the LV pyruvate signal across 106 datasets. To ensure a meaningful comparison, an analysis framework is employed to assess how each model responds to voxel selection and how this affects the correlation with downstream metabolic outcome. The red curve represents the proposed model, which applies α‐thresholding from 1% to 63% (right to left on the correlation plot). For each threshold, Pyr(α(%)) is calculated using Equation (3), and its correlation with Lac+Bic is assessed as Corr(B/Pyr(α(%)), Lac+Bic). In contrast, the green curve represents the traditional percentile‐based model, which selects the top T% of voxels based on signal intensity, ranging from 10% to 100% (left to right on the correlation plot) [3, 16, 21, 38, 43]. For each threshold, Pyr(T(%)) is computed by including the top T% of high‐intensity voxels, and the corresponding correlation is calculated as Corr(B/Pyr(T(%)), Lac+Bic). As the T% threshold increases, the traditional model includes more voxels, which results in a progressive decrease in the average pyruvate signal due to the inclusion of low‐SNR voxels. For example, at *T* = 10%, the average voxel count is 23 ± 4 and the average Pyr(T(%)) value is 498.9 ± 336.6, whereas at *T* = 100%, the voxel counts increases to 229 ± 40 and Pyr(T(%)) decreases to 193.3 ± 116.9. The difference between Pyr(50(%)) and Pyr(100(%)) corresponds to a relative change of 64%, with an effect size of Cohen's *d* = 0.75, suggesting a substantial loss of signal intensity with increased voxel inclusion. Similarly, Pyr(10(%)) differs from Pyr(50(%)) by 58%, with a moderate effect size of Cohen's *d* = 0.66. These findings indicate that the threshold selection has a significant impact on both the extracted signal and its statistical separability (Table S4).

To assess how these differences in Pyr(T(%)) impact metabolic correlation, the green curve in Figure 7 plots the correlation between B/Pyr(T(%)) and Lac+Bic, as a function of *T*. This analysis shows a strong monotonic increase in correlation as voxel count decreases, with a Spearman ρ = 0.85, a pronounced linear trend ($R^{2}$ = 0.79), and a slope of 0.00015. This pattern indicates that the apparent improvement in correlation from 100% (right) to 10% (left) is largely driven by excluding low‐SNR voxels, which narrows the ROI toward the signal‐dense core. The minimum correlation (Corr = −0.593) occurs at just 69 voxels (*T* = 30%), highlighting the model's dependence on restrictive voxel selection.

In contrast, the proposed model (red curve) exhibits a weak, slightly negative monotonic trend followed by a plateau, with Spearman ρ = −0.35, $R^{2}$ = 0.0061, and a near‐zero slope of 8.45 $\times 10^{-6}$. This near‐zero dependence on voxel count is a key strength; it indicates that the model's correlation with metabolism is largely insensitive to small changes in ROI size. The proposed approach reaches its strongest negative correlation at 145 voxels (α = 13%, Corr = −0.596), and beyond this point, the correlation values become tightly clustered along the *x*‐axis. This dense packing of points reflects minimal changes in voxel count and suggests that the proposed approach rapidly converges to a stable and robust ROI definition, with reduced variability across thresholds. This stability supports the proposed model's ability to maintain consistent associations with downstream metabolic outcomes.

Overall, Figure 7 highlights a key difference between the two strategies. The percentile‐based approach enhances correlation primarily by aggressively eliminating low‐SNR voxels, but this benefit comes at the cost of high sensitivity to the exact threshold: the strongest correlation is achieved only within a narrow, restrictive ROI, and the model provides no principled guidance on where noise begins to dominate, where signal peaks, or where stability emerges. In contrast, the proposed model reaches slightly higher maximal correlation at a larger ROI and then enters a broad plateau in which both voxels count, and correlation change only minimally across thresholds. This plateau separates a low‐α noise‐dominated region from a well‐defined optimal α range, and a stable high‐α regime, providing a data‐driven rule for ROI selection. Thus, compared with the traditional model, the proposed model offers a clearer interpretation of noise versus signal, reduced dependence on arbitrary thresholds, and more robust, reproducible correlations with Lac+Bic across datasets.

## 4 Discussion

Accurately quantifying the HP [1‐^13^C]pyruvate signal remains a challenge in metabolic imaging due to variable polarization levels, fast hyperpolarized spin relaxation, and inter‐subject variability in pyruvate delivery [14, 15, 16, 53, 77]. Traditional methods for pyruvate estimation in HP^13^C‐CMR often rely on manual thresholding, anatomical segmentation using proton MRI, or direct segmentation on HP^13^C‐CMR images to define the LV blood pool region [21, 27, 30, 31, 43, 78]. However, these approaches face limitations that affect accuracy and reproducibility. A major challenge is the mismatch between proton and carbon imaging resolutions, which makes it difficult to precisely align the LV blood pool ROI. Additionally, different cardiac and respiratory motion phases between proton scanning and carbon imaging can shift the LV blood pool location, introducing errors in blood pool localization [42]. Direct segmentation on HP^13^C‐CMR images without anatomical reference can also lead to an overestimation of pyruvate signals, reducing the precision of metabolic quantification [21, 42, 43].

To address these challenges, some studies have adopted more data‐driven masking strategies. Mammoli et al. [24] proposed a combined SNR‐ and error‐based voxel inclusion, discarding voxels with low pyruvate or lactate SNR and those with large fitting uncertainty. This improved voxel‐wise kinetic modeling by avoiding poorly fit regions and was more robust than arbitrary cutoffs. However, removing low‐SNR voxels reduces spatial coverage and introduces threshold‐dependent variability, which can compromise the consistency of metabolic maps across heterogeneous datasets. Similarly, Kim et al. [36] applied patch‐based denoising to improve SNR and voxel coverage. This helped improve the quality of metabolic maps like pyruvate‐lactate and pyruvate‐bicarbonate conversion. However, this method depends on parameter tuning and may blur boundaries or bias low‐SNR regions. Sushentsev et al. [38] employed a total carbon SNR maps–based segmentation approach with a fixed SNR threshold (e.g., SNR = 5.0) to define metabolically active tumor cores in prostate cancer. Lee et al. [79] further exemplified threshold dependency by employing hard SNR cutoffs for voxel inclusion (pyruvate = 50, lactate = 20, alanine = 10), which may systematically exclude physiologically relevant voxels in lower‐SNR regions such as deeper organs or areas affected by field inhomogeneity or respiratory motion. Overall, these studies highlight a critical tension in ROI definition for HP^13^C imaging, the need to balance voxel‐wise modeling robustness with spatial inclusiveness and reproducibility. Fixed‐threshold methods can introduce bias and extra variability; while denoising or SNR‐gating approaches improve signal clarity, often reduce anatomical detail, and make results harder to compare across studies.

To overcome these limitations, we developed a distribution‐based thresholding formula for the pyruvate signal systematically accounts for voxel‐wise signal heterogeneity, improving LV pyruvate signal estimation within the LV blood pool ROI. By showing signal distribution patterns and minimizing the impact of low SNR voxels, this method leads to more reliable quantification, enhancing the accuracy of the correlation between pyruvate delivery and metabolic outcome. By modeling the spatial distribution of the pyruvate signal using an exponential fit, the approach objectively identifies and excludes low SNR voxels. As defined in Equation (3), Pyr(α(%)) is computed within the LV blood pool ROI in a case‐adaptive manner, determined by $I_{\max}$ and $I_{\min}$ for each case, rather than applying a fixed threshold or exclusion rule, enabling us to analyze different qualities of HP studies.

Furthermore, the use of a correlation‐based analysis allows identification of an optimal threshold range and detection of the area of changing correlation behaviors and stable range, based on the signal's spatial characteristics, rather than relying on arbitrary cutoff values. Compared to traditional detection models, our approach demonstrated more robust and consistent LV pyruvate estimation. As shown in Figure 6A, the numerical fitting model exhibited a stronger and more stable correlation, consistently performing within the α = 13%–25% range, suggesting that minimizing noise interference allows the model to better capture the metabolic relationship. Beyond thresholds of ∼20%, the correlation with the metabolic signal first declines gradually and then more sharply as fewer voxels remain above the threshold. This shows that a small number of very high‐intensity voxels alone does not reliably represent the true LV pyruvate signal. Indeed, examining the peak‐intensity‐based model, it is vulnerable to extreme voxel intensities and noise, making it less reliable for pyruvate estimation. Further supporting this notion, Figure 7 highlights the inconsistency of a traditional pyruvate estimation that relies on intensity‐based voxel selection. In contrast, the exponential fitting model proposed selects voxels based on a case‐adaptive manner rather than extreme intensity values and demonstrates increased reliability across different datasets.

It is important to emphasize that the main limitation of the percentile‐based approach is not its inability to yield high correlations with metabolic outcome; indeed, it is entirely possible to select a threshold that aligns with the optimal range. However, this model provides no systematic framework to identify where such an optimal threshold lies, where the ROI becomes noise‐dominated, or where stability emerges. Furthermore, it offers no mechanism for verifying whether the selected ROI is physiologically meaningful or reproducible across subjects. As shown in Figure 7, achieving high correlation requires excluding a large number of voxels, resulting in a narrowly focused ROI that may miss a broader physiological context. This highlights a key limitation of the percentile approach: achieving the maximum correlation requires excluding ∼70% of the voxels, thereby discarding a large fraction of voxels that carry useful information about the system dynamics. In contrast, the proposed model reaches its maximum correlation at α = 13%, preserving a more representative set of LV blood pool pyruvate voxels. Also, the proposed model scans across thresholds and explicitly detects these regimes: a low‐threshold range (from 1% to ∼10% thresholding) dominated by noise area, an intermediate range where correlation with metabolism is maximized (optimal ROI) (from 13% to ∼25% thresholding), and a high‐threshold range where the metric stabilizes (≥ 30% thresholding). Moreover, our approach offers a systematic framework for defining the LV blood pool signal, one that is directly informed by the underlying signal distribution and quality of the HP study, and its correlation to metabolic readouts. These comparisons highlight the methodological advantages of our approach: improved consistency, reduced reliance on arbitrary decisions, and enhanced reproducibility across studies. This is particularly critical in the context of test–retest studies, clinical translation, and cross‐subject analyses where robustness and standardization are essential.

In the exponential model (Equation 2), $I_{\max}$ changes only the magnitude of the curve; B value determines how concentrated or diffuse the LV pyruvate signal is within the LV Endo ROI. Larger B values produce a steep fall‐off and a focused, high‐contrast LV pyruvate signal, whereas smaller B values produce a broad, low‐contrast pattern. Because Lac+Bic are generated from pyruvate delivered through this LV pyruvate signal, understanding the spatial distribution of the pyruvate signal is critical. Thus, B value provides a quantitative measure of how well‐formed the LV pyruvate signal is, rather than just how large it is, and is expected to be a more informative descriptor than $I_{\max}$.

Importantly, these relationships were consistent across all three statistical strategies (single‐scan, mixed‐effects, and paired (V1–V2) analyses with Bonferroni correction), indicating robust associations [71, 73, 74, 75].

Despite the improvements offered by this approach, the study has certain limitations related to imaging setup and study design. The ^13^C transmit/receive coil used in this study is optimized for patients with a chest circumference of ∼18–25 cm, making it less suitable for larger or smaller patients due to B_0_ and B_1_^+^ field inhomogeneity and coil sensitivity variations, which can impact signal detection and quantification [21, 43]. Additionally, variability in the delay between pyruvate dissolution and injection may contribute to the variability in polarization levels and signal acquired across subjects, adding complexity to accurately estimating the pyruvate signal across different individuals.

## 5 Conclusion

In this study, we proposed a mathematical model using exponential fitting based on the spatial HP [1‐^13^C]pyruvate signal distribution in the LV blood pool that significantly enhances the quantitative assessment of the LV pyruvate signal. This model generates a numerical fitting value B, that effectively balances the preservation of significant signals and the exclusion of low SNR regions, enabling the development of a thresholding formula to systematically determine the relevant signals within the LV blood pool ROI. Using the exponential numerical fitting value to characterize the LV blood pool HP [1‐^13^C]pyruvate, we demonstrated a strong correlation with the metabolic outcome, the summed [1‐^13^C]lactate and [^13^C]bicarbonate signal, as a solution for pyruvate signal normalization in dynamic nuclear polarization studies.

## Funding

This work was supported by National Center for Advancing Translational Sciences, UL1 TR003163; Cancer Prevention and Research Institute of Texas, RP180404, RR240015.

## Conflicts of Interest

The authors declare no conflicts of interest.

## Supporting information


**Table S1:** CTOX trial investigators.
**Table S2:** Parameter‐wise comparisons between V1 Inj1 and the pooled cohort.
**Table S3:** Summarize correlation B/Pyr (20(%)) with Lac+Bic.
**Table S4:** Summary of LV Blood Pool ROIs from 106 in vivo studies (percentile‐based method).
**Table S5:** Left ventricular blood pool parameters (LV Endo ROI), 106 individual studies.
**Figure S1:** Illustrates the experimental setup for hyperpolarized ^13^C imaging, including: (A) a ^13^C transmit/receive Helmholtz loop‐pair coil, (B) a thermal torso phantom setup, (C) spectral signal acquisition for RF calibration, (D) a thermal 5 M [1‐^13^C]urea phantom used for coil calibration, (E) axial view of the heart, showing the urea phantom positioned centered on top of the upper coil, also the AP distance, sagittal view of the heart, showing the horizontal distance of the urea phantom from the heart's center, and sample [1‐^13^C]urea MRS spectrum.
**Figure S2:** Sorted HP^13^C CMR signal intensities plot against voxel number, demonstrating an exponential pattern. The blue dashed line represents the exponential fitting.
**Figure S3:** Comparison of the raw signal with theoretical modeling. Raw signal intensity (red dots), Equation (2) (green line), and numerical exponential fitting (blue dashed line) for different cases with varying numerical B values. The decreasing B values from (A) to (D) indicate different characteristic signal distributions across voxels in the LV ROIs.
**Figure S4:** Sorted signal intensities and time‐intensity curves. (A) Sorted LV Endo ROI signal intensities with the 20% pyruvate thresholding (dashed blue line). Yellow dots represent the original ROI, and blue dots indicate the selected voxels. (B) Time‐intensity curve for the 20% pyruvate thresholding ROI. (C) Similar analysis for another study shows thresholding adaptability. (D) Time‐intensity curve for the second study, demonstrating a consistent signal pattern.
**FIGURE S5:** Comparison of two LV pyruvate signal models across 106 studies. (A) B/Pyr(α(%)) model, and (B) $I_{\max}/\mathrm{Pyr}\left(\alpha \left(\%\right)\right)$ model. Bic, bicarbonate; Lac, lactate; Pyr, pyruvate.
**Figure S6:** Mixed‐effects model fit quality as a function of the B/Pyr(α(%)). The red curve shows the residual standard deviation (Residual STD) of the random‐intercept mixed effects model for α = 1%–63%. The residual STD reaches its minimum around α = 13%, indicating that this point gives the tightest mixed effects fit between B/Pyr(α(%)) and Lac+Bic.
**Figure S7:** Paired (V1–V2) (Inj1) analysis across 63 for B/Pyr(α(%)). For each α, a paired Δ model is fitted, and the Bonferroni‐corrected *p*‐value is expressed as log_10_(1/p_Bonf_). The blue curve shows that the correlation between ΔB/Pyr(α(%)) and Δ(Lac+Bic) is highly significant for all thresholds, with the strongest evidence (smallest p_Bonf_) around α = 13%.

## Data Availability

The example codes are available on https://github.com/cor‐utsw/hyperpolarized‐13C.
