# Supplementary material for: Optimized Detection of Left Ventricular Hyperpolarized [1‐13C]Pyruvate Signal in Human Cardiac Metabolic Imaging
Source: Magn Reson Med. 2026 Mar 6;96(1):420–34. doi: 10.1002/mrm.70332 (PMC13156456; doi:10.1002/mrm.70332)
Supplement: Supplementary file 1 — Table S1: CTOX trial investigators. Table S2: Parameter‐wise comparisons between V1 Inj1 and the pooled cohort. Table S3: Summarize correlation B/Pyr (20(%)) with Lac+Bic. Table S4: Summary of LV Blood Pool ROIs from 106 in vivo studies (percentile‐based method). Table S5: Left ventricular blood pool parameters (LV Endo ROI), 106 individual studies. Figure S1: Illustrates the experimental setup for hyperpolarized 13C imaging, including: (A) a 13C transmit/receive Helmholtz loop‐pair coil, (B) a thermal torso phantom setup, (C) spectral signal acquisition for RF calibration, (D) a thermal 5 M [1‐13C]urea phantom used for coil calibration, (E) axial view of the heart, showing the urea phantom positioned centered on top of the upper coil, also the AP distance, sagittal view of the heart, showing the horizontal distance of the urea phantom from the heart's center, and sample [1‐13C]urea MRS spectrum. Figure S2: Sorted HP13C CMR signal intensities plot against voxel number, demonstrating an exponential pattern. The blue dashed line represents the exponential fitting. Figure S3: Comparison of the raw signal with theoretical modeling. Raw signal intensity (red dots), Equation (2) (green line), and numerical exponential fitting (blue dashed line) for different cases with varying numerical B values. The decreasing B values from (A) to (D) indicate different characteristic signal distributions across voxels in the LV ROIs. Figure S4: Sorted signal intensities and time‐intensity curves. (A) Sorted LV Endo ROI signal intensities with the 20% pyruvate thresholding (dashed blue line). Yellow dots represent the original ROI, and blue dots indicate the selected voxels. (B) Time‐intensity curve for the 20% pyruvate thresholding ROI. (C) Similar analysis for another study shows thresholding adaptability. (D) Time‐intensity curve for the second study, demonstrating a consistent signal pattern. FIGURE S5: Comparison of two LV pyruvate signal models across 106 studies. (A) B/Pyr(α(%)) m [file MRM-96-420-s001.docx]

**Supporting Information**

**Optimization of hyperpolarized left ventricular [1-^13^C]pyruvate signal detection for human cardiac metabolic imaging**

Fatemeh Khashami^1,2^, Ivan E. Dimitrov^2,3^, Maximilian Fuetterer^4^, Stefan Glöggler^2^, Bei Zhang^2^, Egzona Tan^1^, Sebastian Kozerke^4^, Anke Henning^2^, Tarique Hussain^5^, Craig R. Malloy^1,2,6^, Nisha Unni^1,7,8^, Vlad G. Zaha^1,2,7,8^, *CTOX Trial Investigator(s)^*^*

^1^Department of Internal Medicine, University of Texas Southwestern Medical Center, Dallas, TX, USA;

^2^Advanced Imaging Research Center, University of Texas Southwestern Medical Center, Dallas, TX, USA;

^3^Philips, Cambridge, MA, USA;

^4^Institute for Biomedical Engineering, University and ETH Zurich, Zurich, Switzerland;

^5^Department of Pediatrics, University of Texas Southwestern Medical Center, Dallas, TX, USA;

^6^Dallas VA Medical Center, Dallas, TX, USA;

^7^Harold C. Simmons Comprehensive Cancer Center, University of Texas Southwestern Medical Center, Dallas, TX, USA

^8^Parkland Health, Dallas, TX, USA

**^*^ A complete list of the CTOX trial investigators is provided in the Supporting Information available at https://onlinelibrary.wiley.com/**

**Corresponding Author:**

**Vlad G. Zaha, MD, PhD, MBA;** [Vlad.Zaha@UTSouthwestern.edu](mailto:Vlad.Zaha@UTSouthwestern.edu)**; 5323 Harry Hines Blvd, University of Texas Southwestern Medical Center, Dallas, TX 75390-8568, USA**

**Co-Corresponding Author:**

**Fatemeh Khashami, PhD;** [Fatemeh.Khashami@UTSouthwestern.edu](mailto:Fatemeh.Khashami@UTSouthwestern.edu)

| **Contents** | **Page** |
| --- | --- |
| Supplemental Table S1: CTOX trial collaborators | 2 |
| Supplemental Figure S1: Experimental setup for hyperpolarized ¹³C imaging | 3 |
| Supplemental Figure S2: Sorted HP ¹³C CMR signal intensities plot against voxel rank | 4 |
| Supplemental Figure S3: Comparison of raw signal with theoretical modeling | 5 |
| Supplemental Figure S4: Sorted signal intensities and time-intensity curves | 6 |
| Supplemental Figure S5: Comparison of two LV pyruvate signal models across 106 studies | 7 |
| Supplemental Table S2: Parameter-wise comparisons between V1 Inj1 and the pooled cohort | 8 |
| Supplemental Table S3: Summarize correlation $\mathcal{B}$/Pyr(20(%)) with Lac + Bic | 9 |
| Supplemental Figure S6: Mixed-effects model fit quality as a function of the $\mathcal{B}$/Pyr($\alpha(\%)$) | 11 |
| Supplemental Figure S7: Paired V1–V2 (Inj1) analysis across 63 for $\mathcal{B}$/Pyr($\alpha(\%)$) | 12 |
| Supplemental Table S4: Summary of LV Blood Pool ROIs from 106 in vivo studies (percentile-based method). | 14 |
| Supplemental Table S5: Left ventricular blood pool parameters (LV Endo ROI), 106 individual studies. | 15 |

Table S1: CTOX trial investigators.

| **Collaborator** | **Institution** |
| --- | --- |
| Sheeba Cantanelli | Harold C. Simmons Comprehensive Cancer Center, Dallas, TX, USA |
| Isaac Chan | Harold C. Simmons Comprehensive Cancer Center, Dallas, TX, USA |
| Suzanne Cole | Harold C. Simmons Comprehensive Cancer Center, Dallas, TX, USA and Richardson, TX, USA |
| Suzanne Conzen | Harold C. Simmons Comprehensive Cancer Center, Dallas, TX, USA |
| Glenda Delgado | Harold C. Simmons Comprehensive Cancer Center, Dallas, TX, USA  Parkland Health, Dallas, Texas, USA |
| Deborah Farr | Harold C. Simmons Comprehensive Cancer Center, Dallas, TX, USA |
| Joshua Gruber | Harold C. Simmons Comprehensive Cancer Center, Dallas, TX, USA |
| Barbara Haley | Parkland Health, Dallas, Texas, USA |
| Dawn Klemow | Harold C. Simmons Comprehensive Cancer Center, Dallas, TX, USA |
| Jenny Li | Parkland Health, Dallas, Texas, USA |
| Heather McArthur | Harold C. Simmons Comprehensive Cancer Center, Dallas, TX, USA |
| Alka Mallik | Harold C. Simmons Comprehensive Cancer Center, Dallas, TX, USA and Richardson, TX, USA |
| Poorni Manohar | Harold C. Simmons Comprehensive Cancer Center, Dallas, TX, USA |
| Ina Patel | Harold C. Simmons Comprehensive Cancer Center, Dallas, TX, USA  Moncrief Cancer Insittute, Fort Worth, TX, USA |
| Namrata Peswani | Harold C. Simmons Comprehensive Cancer Center, Dallas, TX, USA and Richardson, TX, USA |
| Sangeetha Reddy | Harold C. Simmons Comprehensive Cancer Center, Dallas, TX, USA |
| Navid Sadeghi | Parkland Health, Dallas, Texas, USA |
| Samira Syed | Parkland Health, Dallas, Texas, USA |

**
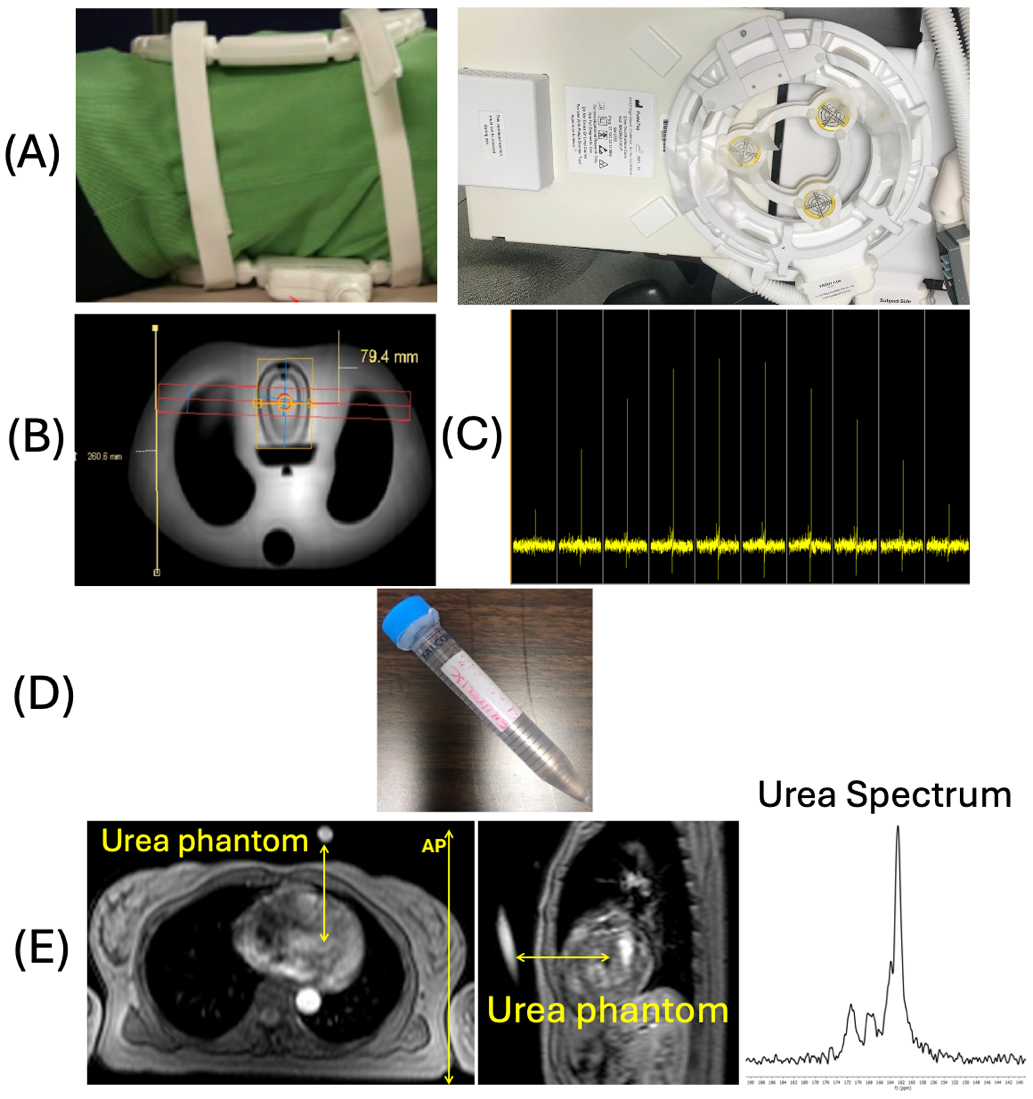
**

Figure S1. Illustrates the experimental setup for hyperpolarized ¹³C imaging, including: (A) a ¹³C transmit/receive Helmholtz loop-pair coil, (B) a thermal torso phantom setup, (C) spectral signal acquisition for RF calibration, (D) a thermal 5M [1-¹³C]urea phantom used for coil calibration, (E) axial view of the heart, showing the urea phantom positioned centered on top of the upper coil, also the AP distance, sagittal view of the heart, showing the horizontal distance of the urea phantom from the heart's center, and sample [1-¹³C]urea MRS spectrum.

To further support the findings presented in this study, additional examples of sorted intensity patterns across multiple *in vivo* datasets are provided in Figure S2.


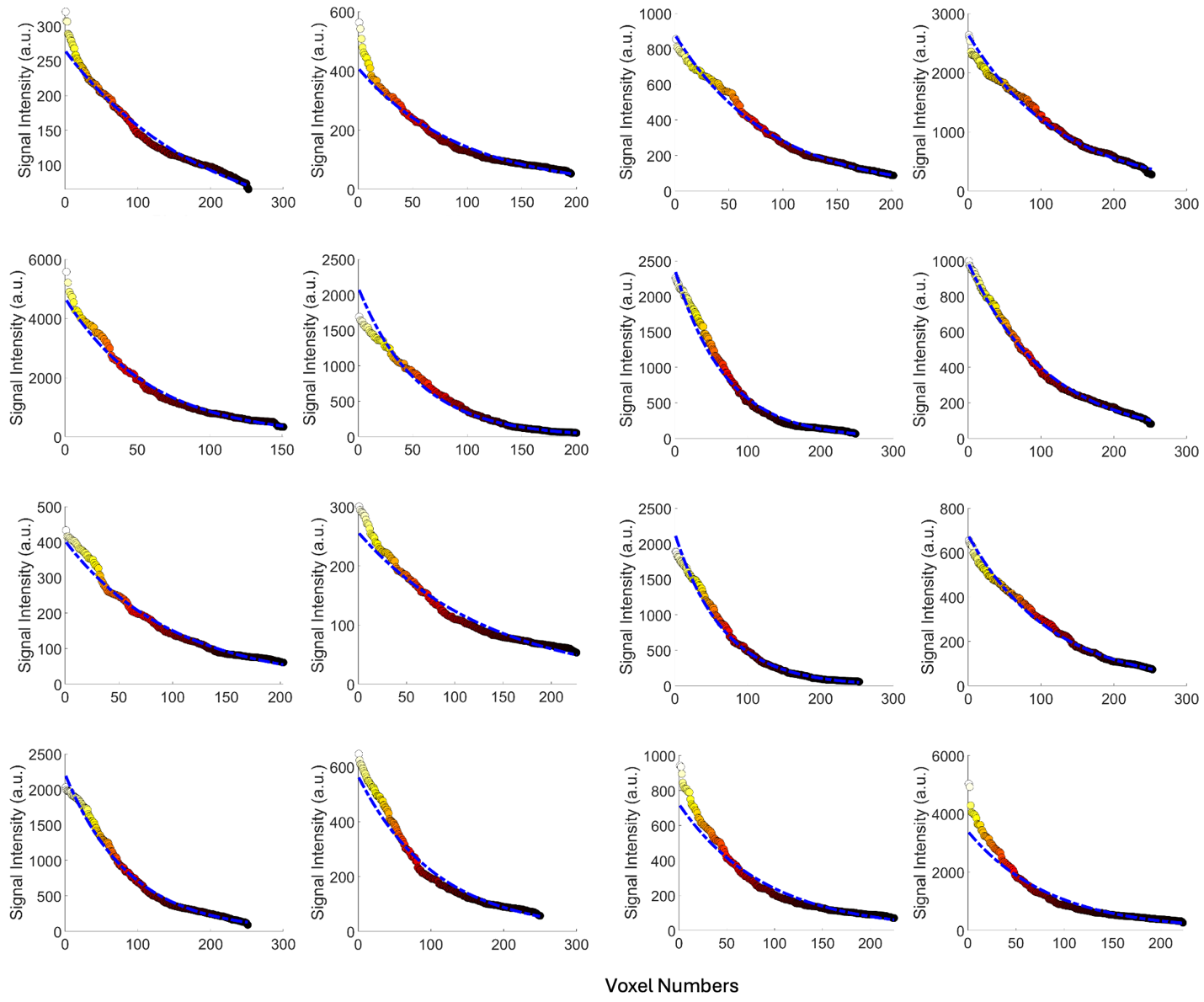


Figure S2. Sorted HP¹³C CMR signal intensities plot against voxel number, demonstrating an exponential pattern. The blue dashed line represents the exponential fitting.

Figure S3 specifically illustrates how the signal intensity varies across voxels, comparing raw data (red dots) with theoretical modeling using Equation 2 (green line) and a numerical exponential fitting (blue dashed line). The decreasing $\mathcal{B}$ values from (A) to (D) reflect different modes of signal distribution in the ROI, demonstrating how signal intensity diminishes at varying rates depending on the numerical $\mathcal{B}$ value. This comparison highlights the effectiveness of $\mathcal{B}$ value in capturing signal patterns and provides a basis for selecting appropriate models for different datasets.


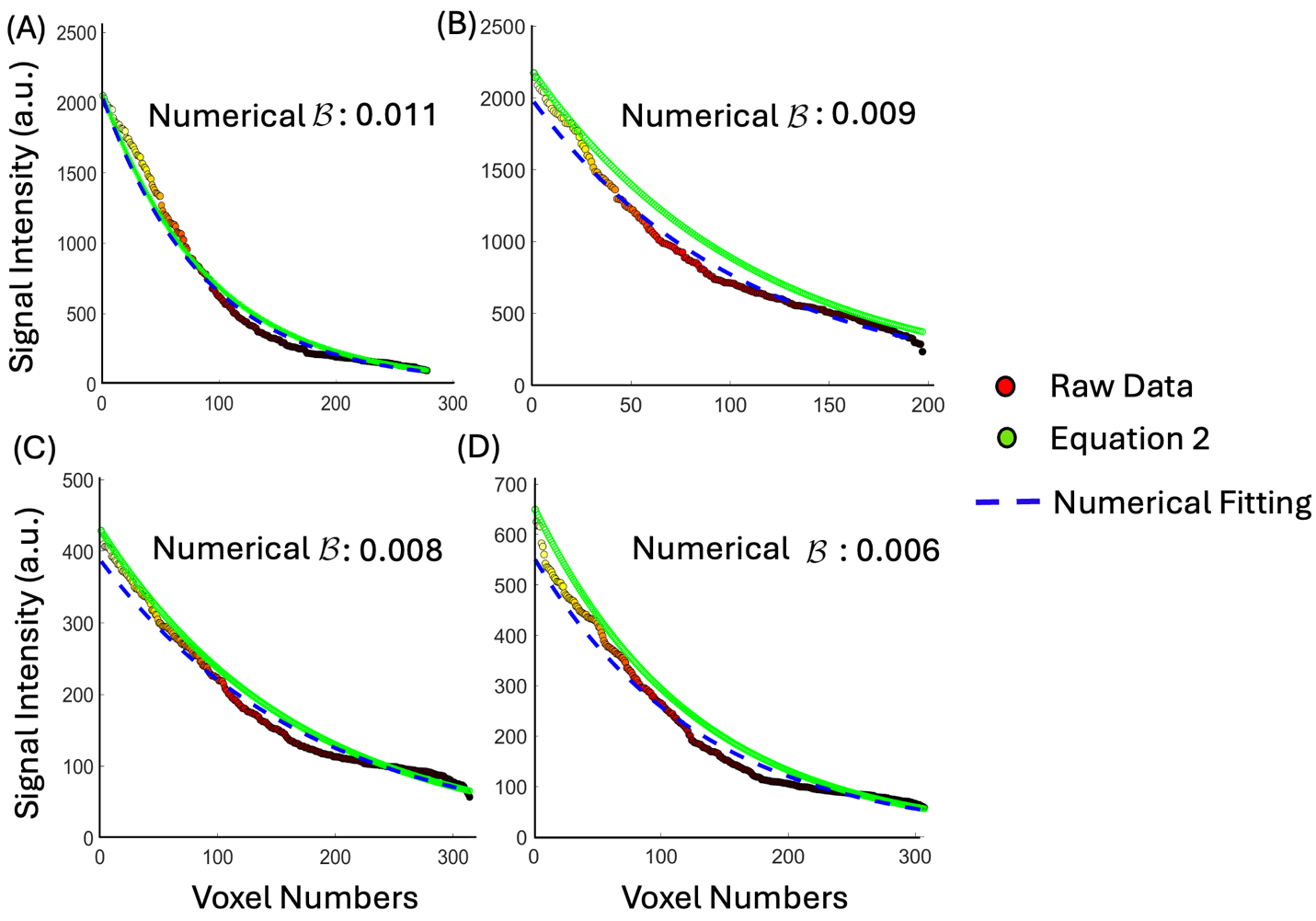


Figure S3. Comparison of the raw signal with theoretical modeling. Raw signal intensity (red dots), Equation 2 (green line), and numerical exponential fitting (blue dashed line) for different cases with varying numerical $\mathcal{B}$ values. The decreasing $\mathcal{B}$ values from (A) to (D) indicate different characteristic signal distributions across voxels in the LV ROIs.

To confirm the effectiveness of the selected threshold, the $20\%$ pyruvate thresholding percentage is applied to two separate HP studies with different (fairly different due to polarization level differences and/or physiological differences in tracer delivery) ranges of signal intensity, as shown in Figure S4. Visualization and analysis are conducted across two HP datasets to support the adaptability of the threshold for identifying relevant regions while minimizing variability. In Figure S4(A), the dashed blue line marks the selected threshold, ensuring that only voxels above $20\%$ of pyruvate thresholding are selected. The remaining voxels after applying the threshold, highlighted as blue dots out of all yellow dots in the HP¹³C CMR image, represent the 20% pyruvate thresholding ROI. The corresponding pyruvate time-intensity curve in Figure S4(B) indicates that the selected ROI preserves the expected signal trend over time. The variation in HP signal profiles, as seen in Figure S4(C) and (D), further supports the threshold's effectiveness in a case of much lower overall pyruvate intensity in isolating relevant regions while maintaining consistency across different datasets.


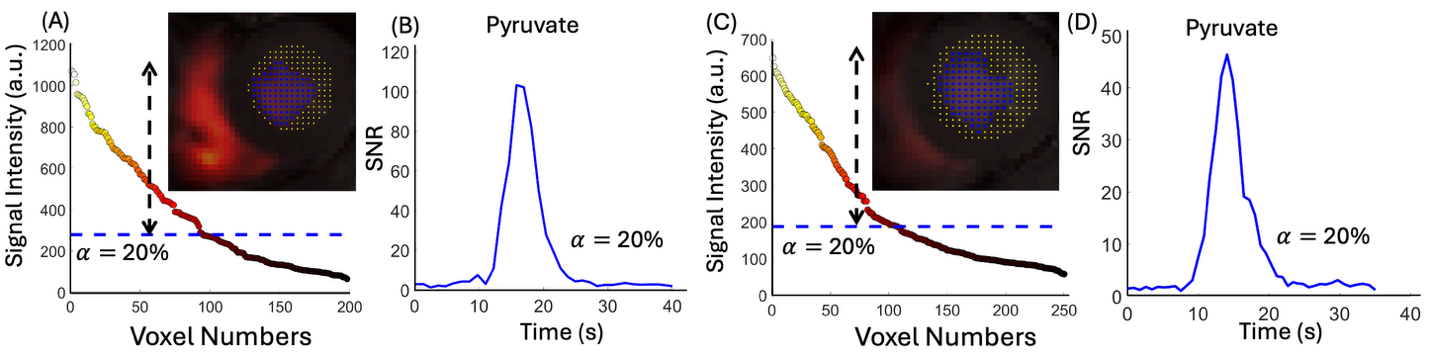


Figure S4. Sorted signal intensities and time-intensity curves. (A) Sorted LV Endo ROI signal intensities with the $20\%$ pyruvate thresholding (dashed blue line). Yellow dots represent the original ROI, and blue dots indicate the selected voxels. (B) Time-intensity curve for the $20\%$ pyruvate thresholding ROI. (C) Similar analysis for another study shows thresholding adaptability. (D) Time-intensity curve for the second study, demonstrating a consistent signal pattern.

The proposed numerical fitting and peak-intensity-based models, $\mathcal{B/}$Pyr$(\alpha($%$))$ and $I_{\max}/$Pyr$(\alpha($%$))$ metrics, can be visualized as three-dimensional (3D) plots for $\alpha$ = 1% to 63% on the x-axis in each study (Figure S5-A and B, respectively). The individual case numbers on the y-axis and the pyruvate metric values on the z-axis offer a comprehensive view of the data distribution across various thresholds and cases. The size of each marker (from larger to smaller) is proportional to the voxel count at that thresholding level. The color code of the plot points (from blue to red) corresponds to Lac+Bic signal intensity extracted from the Mid-Myo ROI, providing a qualitative representation of the metabolic outcome for each case number. For both models, at a low threshold ($\alpha$%$\approx\leq$10%) pyruvate metric exhibits greater variability. As the threshold increases, the pyruvate metric values decrease, reaching stabilization at a relatively large $\alpha($%), when further threshold increases have minimal impact on the pyruvate metric. A comparison of variability across all studies for the pyruvate metric at all threshold values showed the coefficients of variability for $\mathcal{B/}$Pyr$(\alpha($%$))$values are consistently lower than the corresponding ones for $I_{\max}/$Pyr$(\alpha($%$))$(48.75% ± 0.01% vs. 55% ± 0.03%, p-value<0.0001). An effect size analysis using Cohen’s d between $\alpha$ = 1% and $\alpha$ = 20% demonstrates large effects for both models: 0.91 for $\mathcal{B/}$Pyr$(\alpha($%$))$ and 0.82 for $I_{\max}/$Pyr$(\alpha($%$))$. These analyses support the qualitative impression that the numerical fitting model is providing a more homogenous LV pyruvate signal model compared to the peak-intensity-based model, while both present a substantial effect of the thresholding.


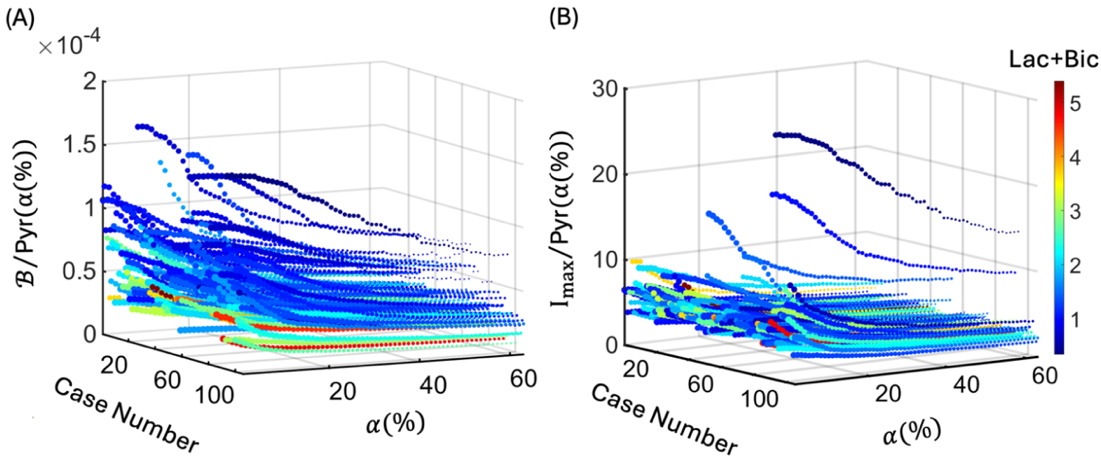


FIGURE **S5.** Comparison of two LV pyruvate signal models across 106 studies. (A) $\mathcal{B}/$Pyr$(\alpha($%$))$ model, and (B) $I_{\max}/Pyr(\alpha(\%))$ model. Abbreviations: Pyr, pyruvate; Bic, bicarbonate; Lac, lactate.

1. **Statistical Analysis**

This study includes repeated scans from the same subjects across different visits and injections (e.g., V1/V2, Inj1/Inj2), where V1 represents the pre-treatment visit and V2 the post-treatment one. These repeated measurements may not statistically be independent, as they sample the same heart under similar physiological and imaging conditions. This may introduce subject-specific sources of variation that, if ignored, may lead to underestimation of variability and overstatement of effect sizes or correlations. Therefore, we analyzed whether the repeated injection can result in within-subject correlations. We adopted a statistical framework explicitly accounting for this within-subject dependency using mixed-effects^1-5^ and paired analysis models to avoid biased inferences and ensure valid comparisons^6-8^. We showed that the main conclusions remained unchanged.

To address these concerns, we first tested whether visit/injection subgroups could be treated as a single pooled cohort, (i) restricting the dataset to pre-treatment scans and using only the first injection for subjects with two injections. This uniform rule prevented duplication and reduced bias from selection or physiological carryover. We then explicitly modeled (ii) within-subject correlation using both mixed-effects and (iii) paired pre–post approaches. All analyses were performed for $\mathcal{B/}$Pyr$(\alpha($%$))$, as part of the proposed framework. Linear mixed-effects models with a random subject intercept were used to include all injections while accounting for within-subject correlation, and a complementary paired (V1–V2) analysis (for Inj1 only) focused purely on within-subject changes. Because the correlation between $\mathcal{B/}$Pyr$(\alpha($%$))$and Lac + Bic was evaluated across 63 thresholds, p-values from the paired models were adjusted using a Bonferroni correction to control the family-wise error rate.

1. **Parameter-wise Comparisons Between Subgroups and the Pooled Cohort:**

Following the model setup mentioned above, we examined how each visit/injection subgroup compared to the overall cohort of 106 scans (noted as REF in Table S2). For this plan, we first tested whether the visit/injection subgroup could be treated as a single pooled cohort, restricting the dataset to V1 Inj1 for subjects with two injections. V1 Inj1 (n = 49) were compared across key parameters, including $\mathcal{B}$ value, Lac + Bic, $I_{\max}$, and voxel count ($\mathcal{N}$). Parameter-wise comparisons between V1 Inj1 and the pooled cohort are summarized in Table S2.

For $\mathcal{B}$ value, subgroup average percentage difference (subgroup-REF) is ~3% of the overall mean, with minimal Fisher Z-test^1^, |Z|-score ≤ 0.09, suggesting that $\mathcal{B}$ value signals are effectively identical across visits and injections. These minor shifts (~3%) reflect small differences in the number of voxels included in the ROI, as described in Eq. 3, where $\mathcal{B}$ value and $\mathcal{N}$ are inversely related. To support, the voxel count varied by about $-$3.5% from the cohort average (228 voxels), with |Z|-score 0.20. Lac + Bic values are ~$-$5% lower in V1 Inj1 compared to the pooled mean, but the standardized difference is still very small (|Z|-score ~0.09). Peak intensities ($I_{\max}$) exhibited a similar pattern, V1 Inj1 showing slight decreases relative to the 106-scan mean ~$-$3%, with |Z|-score ~0.04. In summary, across all parameters, subgroup-to-pooled standard deviation ratios (STD_ratio_) were close to 1, indicating that V1 Inj1 is not materially different from the pooled cohort in any parameter, which supports treating the 106 scans as a single cohort in the main analyses, while explicitly modeling repeated measures as described below.

Table S2: Parameter-wise comparisons between V1 Inj1 and the pooled cohort

| Subgroup | Parameter | Mean  Subgroup | Mean  REF | Diff (mean)  (Subgroup-REF) | Percent Diff  (%) | Z score | STD_subgroup_ | STD_REF_ | STD_ratio_ |
| --- | --- | --- | --- | --- | --- | --- | --- | --- | --- |
| V1 Inj1, n = 49 | $\mathcal{B}$ value | 0.01085 | 0.01054 | 0.00031 | 2.9 | 0.09 | 0.0037 | 0.0037 | 0.99 |
| REF, n = 106 | $\mathcal{B}$ value | 0.01054 | 0.01054 | 0 | 0 | 0 | 0.0037 | 0.0037 | 1 |
|  |  |  |  |  |  |  |  |  |  |
| V1 Inj1, n = 49 | Lac + Bic | 1.75 | 1.84 | -0.09 | -4.8 | -0.09 | 0.96 | 0.98 | 0.98 |
| REF, n = 106 | Lac + Bic | 1.84 | 1.84 | 0 | 0 | 0 | 0.98 | 0.98 | 1 |
|  |  |  |  |  |  |  |  |  |  |
| V1 Inj1, n = 49 | $I_{\max}$ | 1251 | 1287 | -36.3 | -2.8 | -0.04 | 960 | 947 | 1.01 |
| REF, n = 106 | $I_{\max}$ | 1287 | 1287 | 0 | 0 | 0 | 947 | 947 | 1 |
|  |  |  |  |  |  |  |  |  |  |
| V1 Inj1, n = 49 | $\mathcal{N}$ | 220 | 228 | -8 | -3.5 | -0.2 | 40 | 40 | 1 |
| REF, n = 106 | $\mathcal{N}$ | 228 | 228 | 0 | 0 | 0 | 40 | 40 | 1 |
|  |  |  |  |  |  |  |  |  |  |

To verify that the relationship between normalized $\mathcal{B}$/Pyr($\alpha(\%$)) and metabolism is stable across visits and injections, we compared the correlation V1 Inj1 subgroup with the pooled cohort (Table S3). The reference correlation for all 106 scans (e.g., $\alpha$ = 20%) is −0.59, and subgroup correlation is −0.61 (V1 Inj1), showing the subgroup values clustered tightly around the reference (|Diff (Corr)| ≤ 0.027). The resulting test statistics are small (|Z| ≤ 0.2, and large p-value 0.8), indicating no evidence that the correlation $\mathcal{B}$/Pyr(20(%)) and Lac + Bic differs between V1 Inj1 and all 106 scans. Thus, $\mathcal{B}$/Pyr(20(%)) exhibits a stable, moderately strong negative correlation with Lac + Bic across visit and injections.

Table S3: Summarize correlation $\mathcal{B}$/Pyr (20(%)) with Lac + Bic

| Subgroup | Parameter | Subgroup | REF | Diff (Corr)  (Subgroup-REF) | Percent Diff  (%) | Z score |
| --- | --- | --- | --- | --- | --- | --- |
| V1 Inj1, n = 49 | Corr ($\mathcal{B}$/Pyr (20(%), Lac + Bic) | -0.61 | -0.59 | -0.027 | 0.04 | -0.2 |
| REF, n = 106 | Corr ($\mathcal{B}$/Pyr (20(%), Lac + Bic) | -0.59 | -0.59 | 0 | 0 | 0 |

1. **Within-Subject Correlation and Linear Mixed-Effects Models:**

Several subjects contributed more than one scan (e.g., V1/V2 and Inj1/Inj2), so individual injections from the same subject may not be independent observations. To account for this within-subject correlation while still using all 106 injections, a linear mixed-effects model with a random intercept per subject is fitted as ${(Lac + Bic)}_{ij}$ $=$ $\beta₀$ + $\beta₁ {\mathcal{B}/Pyr(\alpha\left( \% \right))}_{ij}$ + $u_{i}$ + $\varepsilon_{ij}$ , where $i$ indexes subjects and $j$ indexes injections^2-3^. In this formulation, the random intercept $u_{i}$ captures subject-specific baseline differences in overall metabolic level between hearts, and $\beta₁$ is a single population-level slope that quantifies the correlation between $\mathcal{B}$/Pyr($\alpha(\%)$) and Lac + Bic after adjusting for these subject effects. $\beta₀$ is the fixed intercept representing the average baseline Lac + Bic level. The residual term $\varepsilon_{ij}$ represents within-subject variability around this relationship. Because repeated scans from the same subject share the same random intercept, the model naturally handles correlated measurements rather than treating each injection as independent.

For each $\mathcal{B}$/Pyr($\alpha(\%)$) threshold $\alpha$ = 1…63%, a separate mixed-effects model of this form is fitted. Across all thresholds, the estimated fixed-effect slopes are negative and become more negative as $\alpha$ increases (slope is from roughly $-2.0\times10⁴$ at $\alpha$ = 1% to about $-4.5\times10⁴$ at $\alpha$ = 63%), with Bonferroni-corrected p-values on the order of $10⁻¹^{3}-10⁻¹⁴$ (all≪0.001). Thus, even after accounting for repeated scans from the same subject, higher $\mathcal{B}$/Pyr($\alpha(\%)$) is consistently correlated with lower Lac + Bic at all thresholds from 1% to 63%, showing a stable negative relationship that is not driven by any single $\alpha$ value.

To summarize how model performance varies with the threshold, we computed the residual standard deviation (residual STD) (square root of the residual variance) for each $\alpha$ and plotted versus $\alpha(\%)$ (Figure S6). A smaller residual STD indicates a more effective and predictive mixed-effects model. The residual STD shows a clear minimum $\alpha$ ≈ 13%, indicating that this point yields the tightest mixed effects fit between $\mathcal{B}$/Pyr($\alpha(\%$)) and Lac + Bic. In other words, while the negative correlation is strong for all thresholds, the mixed-effects analysis identifies $\alpha$ ≈ 13% as a data-driven “optimal” range for defining the LV pyruvate blood pool ROI. This finding aligns with the observation of the minimum point in Figure 6A. Furthermore, we are able to see that the curve decreases for $\alpha$ values below ≈13% and increases for $\alpha$ values above this point, indicating a noise-dominated regime at low $\alpha$, where the residual STD is high and falls as noisy voxels are excluded, and a signal-limited regime at higher $\alpha$ (e.g., >30%), where the residual STD increases again as too many informative voxels are removed.


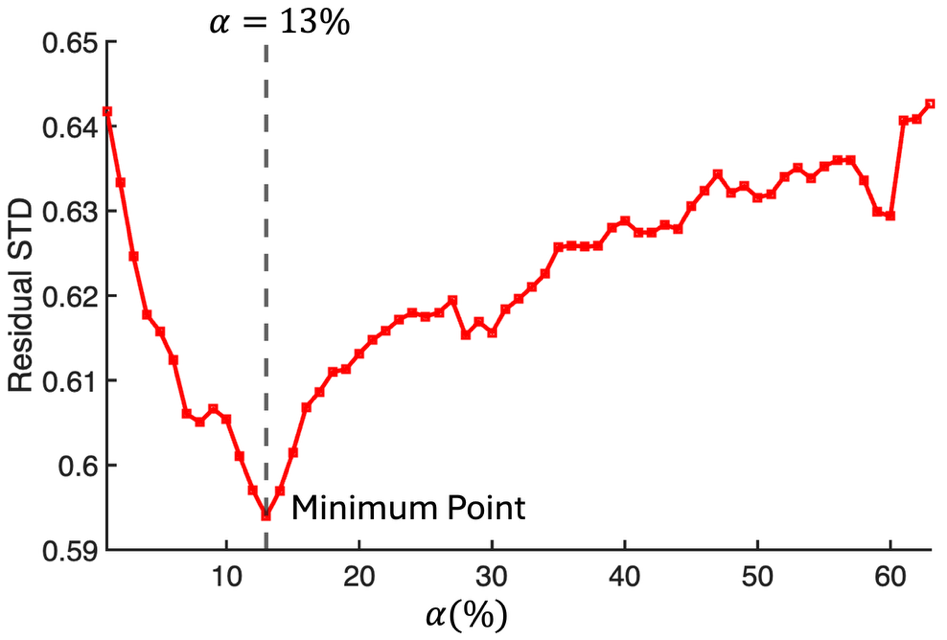


Figure S6: Mixed-effects model fit quality as a function of the $\mathcal{B}$/Pyr($\alpha(\%)$). The red curve shows the residual standard deviation (Residual STD) of the random-intercept mixed effects model for $\alpha$ = 1–63%. The residual STD reaches its minimum around $\alpha$ ≈ 13%, indicating that this point gives the tightest mixed effects fit between $\mathcal{B}$/Pyr($\alpha(\%)$) and Lac + Bic.

1. **Within-Subject Correlation, Paired Pre–Post Analysis, and Bonferroni Correction:**

As a complementary approach focused purely on within-subject change, a paired pre–post analysis is performed using Inj1 data only. For subjects with both V1 and V2 scans (n = 38, number of data points used in each regression), visit-to-visit differences are computed as Δ-variables: Δ$\mathcal{B}$/Pyr($\alpha(\%)$) = $\mathcal{B}$/Pyr($\alpha(\%)$)_V2_$-$ $\mathcal{B}$/Pyr($\alpha(\%)$)_V1_, Δ(Lac + Bic) = (Lac + Bic)_V2_ $-$ (Lac + Bic)_V1_. This Δ-based regression is one of the standard approaches for analyzing pre–post data, alongside ANCOVA and mixed models^4-5^.

For each $\alpha$ = 1…63% (number of separate regressions), a simple linear regression of Δ(Lac + Bic) on Δ$\mathcal{B}$/Pyr($\alpha(\%)$) is fitted^6-7^. We model the paired changes as Δ(Lac + Bic) = $\beta₀$ + $\beta₁$ Δ$\mathcal{B}$/Pyr($\alpha(\%)$) + $\varepsilon$, where $\beta₀$ is the intercept, $\beta₁$ is the slope (how much Δ(Lac + Bic) changes per unit change in Δ$\mathcal{B}$/Pyr($\alpha(\%)$); all slopes are negative with error), $\varepsilon$ is the error term represents the residual STD. We also computed $R^{2}$, the fraction of variance in Δ(Lac + Bic) explained by Δ$\mathcal{B}$/Pyr($\alpha(\%)$). For each $\alpha$, we fit one regression using all 38 subjects. The same correlation is evaluated at 63 thresholds, p-values are adjusted with a Bonferroni correction^6-8^ (p_Bonf_ = min(p-value × 63, 1)) to control the family-wise error rate.

In this paired analysis, all slopes are negative, ranging from $-2.6\times10⁴$ at $\alpha$ = 1% to $-5.3\times10⁴$ at $\alpha$ = 63%. The uncorrected p-values are on the order of $10⁻⁸-10⁻⁹$ and remain highly significant after Bonferroni correction (p_Bonf_ $\lesssim10⁻⁷$). The $R^{2}$ values are consistently high across the entire range of $\alpha$(%), starting around 0.58 at $\alpha$ = 1%, peaking at approximately 0.66 near $\alpha$ = 13%, and gradually declining to ~0.55 by $\alpha$ = 63%. This pattern indicates that the model robustly captures within-subject changes in $\mathcal{B}$/Pyr($\alpha(\%)$) relative to changes in Lac + Bic across a broad range of ROI sizes.

In Figure S7, the paired (V1–V2) (for Inj1) analysis across 63 thresholds is presented. The blue curve shows the Bonferroni-corrected significance level, plotted as log₁₀(1/p_Bonf_), as a function of $\alpha$(%). The curve reaches its most negative value (minimum) at $\alpha$ ≈ 13%, corresponding to the smallest p_Bonf_ and thus the strongest statistical evidence for the paired correlation between Δ$\mathcal{B}$/Pyr($\alpha(\%)$) and Δ(Lac + Bic). As $\alpha$ moves away from this region in either direction, log₁₀(1/p_Bonf_) increases toward less negative values, indicating that p_Bonf_ becomes larger and the (V1–V2) difference becomes less statistically significant, so the strongest paired effect is confined to a narrow band of $\mathcal{B}$/Pyr($\alpha(\%)$) values around 13%.


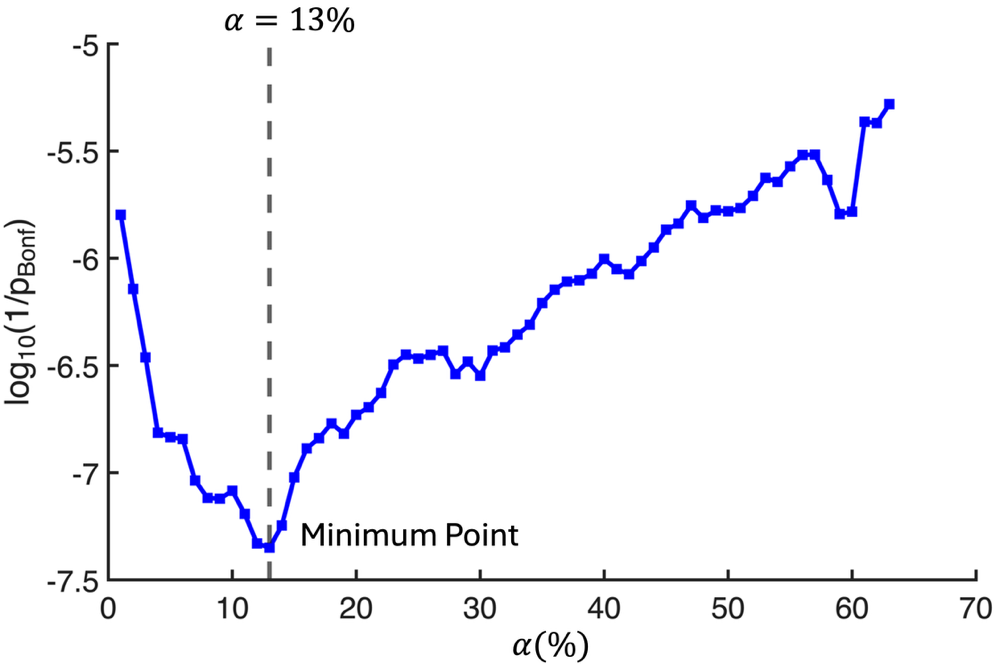


Figure S7: Paired (V1–V2) (Inj1) analysis across 63 for $\mathcal{B}$/Pyr($\alpha(\%)$). For each $\alpha$, a paired Δ model is fitted, and the Bonferroni-corrected p-value is expressed as log₁₀(1/p_Bonf_). The blue curve shows that the correlation between Δ$\mathcal{B}$/Pyr($\alpha(\%)$) and Δ(Lac + Bic) is highly significant for all thresholds, with the strongest evidence (smallest p_Bonf_) around $\alpha$ ≈ 13%.

To have a better understanding, two thresholds are particularly selected. At $\alpha$ = 20%, the slope is $-3.73\times10⁴$ with $R^{2}$ ≈ 0.62, p-value = $2.95\times10⁻⁹$, and p_Bonf_ = $1.86\times10⁻⁷$ (n = 38 pairs). At $\alpha$ = 13%, where $R^{2}$ is maximal, the slope is $-3.34\times10⁴$ with $R^{2}$ = 0.65, p-value = $7.09\times10⁻¹⁰$, and p_Bonf_ = $4.47\times10⁻⁸$. Thus, in a strict paired pre–post design, increases in $\mathcal{B}$/Pyr($\alpha(\%)$) are strongly connected with decreases in Lac + Bic, and this correlation is highly significant even under conservative multiple-comparison correction. The optimum lies in a broad range around $\alpha$ ≈ 13–25%, but thresholds such as $\alpha$ = 20% perform nearly as well. In summary, these analyses show that (i) treating all 106 scans as a single cohort is reasonable because the subgroups are not statistically distinct, and (ii) the key conclusions about the $\mathcal{B}$/Pyr($\alpha(\%)$) and Lac + Bic relationship remain robust when within-subject correlation is modeled explicitly using both mixed-effects and paired pre–post designs with appropriate multiple-comparison control.

**Reference:**

1. Abdi, Hervé. "Z-scores." *Encyclopedia of measurement and statistics* 3 (2007): 1055-1058.
2. Brown, Violet A. "An introduction to linear mixed-effects modeling in R." *Advances in Methods and Practices in Psychological Science* 4, no. 1 (2021): 2515245920960351.
3. Guo, Wensheng. "Functional mixed effects models." Biometrics 58, no. 1 (2002): 121-128.
4. O'Connell NS, Dai L, Jiang Y, Speiser JL, Ward R, Wei W, Carroll R, Gebregziabher M. "Methods for Analysis of Pre-Post Data in Clinical Research: A Comparison of Five Common Methods". J Biom Biostat. 2017 Feb 24;8(1):1-8. doi: 10.4172/2155-6180.1000334. PMID: 30555734; PMCID: PMC6290914.
5. Zhaoxia Yu, Michele Guindani, Steven F. Grieco, Lujia Chen, Todd C. Holmes, Xiangmin Xu, "Beyond t test and ANOVA: applications of mixed-effects models for more rigorous statistical analysis in neuroscience research", 2022, https://doi.org/10.1016/j.neuron.2021.10.030.
6. Shi, Qian, Emily S. Pavey, and Rickey E. Carter. "Bonferroni‐based correction factor for multiple, correlated endpoints." *Pharmaceutical statistics* 11, no. 4 (2012): 300-309.
7. Armstrong RA. "When to use the Bonferroni correction". Ophthalmic Physiol Opt. 2014 Sep;34(5):502-8. doi: 10.1111/opo.12131. Epub 2014 Apr 2. PMID: 24697967.
8. Napierala, Matthew A. "What is the Bonferroni correction?" *Aaos now* (2012): 40-41.

Table S4. Summary of LV Blood Pool ROIs from 106 in vivo studies (percentile-based method).

| $T$(%) | $\mathcal{N}$ (Voxels) | Pyr$(T($%$))$ (a.u.) |
| --- | --- | --- |
| 10 | 23 ± 4 | 498.9 ± 336.6 |
| 20 | 46 ± 8 | 448.2 ± 298.7 |
| 30 | 69 ± 12 | 399.6 ± 262.1 |
| 40 | 92 ± 16 | 355.3 ± 229.6 |
| 50 | 115 ± 20 | 316.2 ± 201.5 |
| 60 | 137 ± 24 | 283.1 ± 178.4 |
| 70 | 160 ± 28 | 254.9 ± 158.7 |
| 80 | 183 ± 32 | 231.1 ± 142.3 |
| 90 | 206 ± 36 | 210.9 ± 128.6 |
| 100 | 229 ± 40 | 193.3 ± 116.9 |

Table S5: Left ventricular blood pool parameters (LV Endo ROI), 106 individual studies.

V1, visit 1 (pre-treatment), V2, visit 2 (post-treatment), Inj1 (injection 1), Inj2 (injection 2)

| Study # | I_max_ | I_min_ | Total pyruvate signal | Number of Voxel | $\mathcal{B}$ numerical value | SNR  Pyruvate | SNR  Lac + Bic |
| --- | --- | --- | --- | --- | --- | --- | --- |
| CTOX022V2 | 320.7 | 65.0 | 36998.0 | 252 | 0.00527 | 49.4 | 1.07 |
| CTOX023V1Inj1 | 563.3 | 52.7 | 34245.1 | 195 | 0.01056 | 88.6 | 0.92 |
| CTOX023V1Inj2 | 401.5 | 46.9 | 34666.1 | 195 | 0.00848 | 101.4 | 0.82 |
| CTOX023V2 | 1464.9 | 99.6 | 83185.4 | 151 | 0.01779 | 225.5 | 2.79 |
| CTOX024V1 | 1143.3 | 91.4 | 69974.9 | 153 | 0.01541 | 217.9 | 1.96 |
| CTOX024V2 | 5576.5 | 331.9 | 257425.7 | 151 | 0.01696 | 542.8 | 3.68 |
| CTOX025V1 | 1690.4 | 53.1 | 108979.2 | 200 | 0.01826 | 379.5 | 1.88 |
| CTOX025V2 | 1223.2 | 56.5 | 87385.0 | 250 | 0.01207 | 156.2 | 2.17 |
| CTOX026V1 | 855.6 | 87.7 | 69291.5 | 202 | 0.01140 | 126.0 | 1.40 |
| CTOX026V2 | 2633.5 | 278.8 | 288995.9 | 252 | 0.00782 | 274.9 | 2.07 |
| CTOX027V1Inj1 | 432.9 | 61.0 | 35616.4 | 203 | 0.00985 | 97.9 | 1.02 |
| CTOX027V1Inj2 | 300.5 | 53.4 | 28645.0 | 226 | 0.00731 | 67.5 | 0.86 |
| CTOX027V2 | 1251.8 | 60.8 | 115196.8 | 222 | 0.01329 | 252.1 | 2.16 |
| CTOX028V1Inj1 | 2258.4 | 65.2 | 164357.6 | 248 | 0.01438 | 349.1 | 1.77 |
| CTOX028V1Inj2 | 998.8 | 81.9 | 97521.5 | 251 | 0.00910 | 131.9 | 0.96 |
| CTOX028V2 | 2031.9 | 92.2 | 184054.2 | 251 | 0.01137 | 266.7 | 2.86 |
| CTOX029V1Inj1 | 647.5 | 57.2 | 55784.0 | 250 | 0.00934 | 87.9 | 1.14 |
| CTOX029V1Inj2 | 888.6 | 68.2 | 79760.3 | 200 | 0.01199 | 207.4 | 2.09 |
| CTOX029V2 | 1889.3 | 57.5 | 139845.9 | 253 | 0.01500 | 244.9 | 3.20 |
| CTOX030V1Inj1 | 655.3 | 73.7 | 68666.8 | 253 | 0.00872 | 134.6 | 1.28 |
| CTOX030V1Inj2 | 965.0 | 72.5 | 112111.9 | 249 | 0.00912 | 267.6 | 1.01 |
| CTOX030V2Inj1 | 1275.9 | 69.1 | 130601.6 | 253 | 0.01183 | 248.0 | 2.90 |
| CTOX030V2Inj2 | 1883.0 | 108.7 | 181004.3 | 249 | 0.01098 | 408.7 | 3.13 |
| CTOX031V1Inj1 | 1637.4 | 127.1 | 135540.4 | 198 | 0.01188 | 273.1 | 1.44 |
| CTOX031V1Inj2 | 1383.4 | 232.2 | 160947.3 | 252 | 0.00603 | 150.0 | 1.22 |
| CTOX031V2 | 866.3 | 87.5 | 69178.5 | 202 | 0.01138 | 125.7 | 1.38 |
| CTOX033V1Inj1 | 251.6 | 38.2 | 21898.1 | 197 | 0.00834 | 48.4 | 0.74 |
| CTOX033V1Inj2 | 554.5 | 45.8 | 40872.3 | 203 | 0.01231 | 115.4 | 0.87 |
| CTOX033V2 | 1903.6 | 78.4 | 128398.9 | 197 | 0.01596 | 310.0 | 3.19 |
| CTOX034V1 | 669.9 | 53.2 | 53991.8 | 247 | 0.01052 | 110.2 | 1.94 |
| CTOX034V2 | 484.4 | 64.0 | 49119.5 | 253 | 0.00703 | 87.5 | 1.83 |
| CTOX035V1 | 1214.7 | 60.0 | 85317.5 | 250 | 0.01250 | 123.5 | 0.87 |
| CTOX035V2 | 1690.5 | 84.5 | 144911.0 | 253 | 0.01218 | 195.6 | 0.91 |
| CTOX036V1 | 357.6 | 70.3 | 38319.4 | 196 | 0.00666 | 72.9 | 1.34 |
| CTOX036V2 | 1021.2 | 59.2 | 107639.8 | 315 | 0.00829 | 160.5 | 2.05 |
| CTOX037V1Inj1 | 1755.7 | 87.2 | 130166.3 | 204 | 0.01508 | 332.3 | 3.67 |
| CTOX037V1Inj2 | 1019.8 | 65.5 | 75136.0 | 200 | 0.01308 | 169.8 | 2.52 |
| CTOX037V2 | 3198.4 | 81.5 | 175748.7 | 202 | 0.01798 | 339.2 | 5.40 |
| CTOX038V1 | 2073.0 | 167.4 | 172427.1 | 251 | 0.00833 | 264.3 | 3.17 |
| CTOX039V1Inj1 | 1070.9 | 63.6 | 73304.6 | 198 | 0.01388 | 212.2 | 1.78 |
| CTOX040V1 | 1129.1 | 118.7 | 77079.5 | 152 | 0.01375 | 224.6 | 1.51 |
| CTOX040V2 | 1920.8 | 81.7 | 177910.4 | 254 | 0.01201 | 293.1 | 2.29 |
| CTOX041V1 | 1608.8 | 217.4 | 139073.7 | 198 | 0.00835 | 226.4 | 1.79 |
| CTOX042V1 | 766.0 | 61.1 | 37285.3 | 152 | 0.01666 | 109.6 | 1.77 |
| CTOX042V2 | 1614.9 | 81.9 | 108451.9 | 198 | 0.01545 | 244.2 | 2.93 |
| CTOX043V1 | 898.3 | 70.5 | 81247.1 | 196 | 0.01321 | 222.8 | 1.19 |
| CTOX043V2 | 1571.0 | 67.1 | 159224.3 | 250 | 0.01167 | 318.4 | 2.60 |
| CTOX044V1 | 2603.4 | 78.9 | 176148.2 | 199 | 0.01835 | 445.6 | 2.27 |
| CTOX045V1 | 1534.8 | 74.1 | 158604.1 | 251 | 0.01059 | 313.2 | 2.69 |
| CTOX046V1 | 496.6 | 67.8 | 65025.7 | 314 | 0.00546 | 89.6 | 1.18 |
| CTOX046V2 | 210.2 | 67.1 | 29194.6 | 249 | 0.00362 | 55.5 | 1.01 |
| CTOX047V1 | 2191.3 | 217.3 | 166678.7 | 199 | 0.01166 | 205.0 | 1.56 |
| CTOX047V2 | 3051.7 | 170.0 | 249206.6 | 253 | 0.01017 | 172.5 | 1.40 |
| CTOX048V1 | 1064.2 | 52.0 | 134261.5 | 311 | 0.00936 | 264.6 | 2.04 |
| CTOX048V2 | 137.3 | 44.8 | 26362.3 | 287 | 0.00286 | 33.3 | 0.77 |
| CTOX049V1 | 1981.1 | 80.5 | 146477.1 | 202 | 0.01541 | 340.3 | 3.92 |
| CTOX049V2 | 1815.8 | 71.5 | 144481.9 | 202 | 0.01479 | 317.8 | 4.28 |
| CTOX050V1 | 1241.0 | 115.5 | 111523.2 | 200 | 0.00982 | 230.8 | 1.49 |
| CTOX050V2 | 1751.6 | 170.7 | 194298.7 | 255 | 0.00628 | 303.3 | 1.72 |
| CTOX051V2 | 1738.5 | 85.9 | 141007.1 | 198 | 0.01559 | 340.8 | 2.84 |
| CTOX052V1 | 215.4 | 74.4 | 24580.2 | 193 | 0.00448 | 41.1 | 0.82 |
| CTOX052V2 | 580.6 | 94.5 | 75466.3 | 310 | 0.00521 | 80.6 | 1.12 |
| CTOX053V1 | 514.9 | 86.1 | 39165.2 | 196 | 0.00901 | 88.1 | 1.39 |
| CTOX053V2Inj1 | 810.5 | 93.7 | 82594.4 | 253 | 0.00841 | 120.0 | 1.95 |
| CTOX054V1 | 341.6 | 43.2 | 25430.2 | 199 | 0.00887 | 54.5 | 1.27 |
| CTOX054V2 | 125.9 | 45.1 | 17436.9 | 223 | 0.00323 | 22.4 | 0.66 |
| CTOX055V1 | 1060.7 | 79.9 | 75868.7 | 251 | 0.01030 | 126.3 | 1.31 |
| CTOX055V2 | 955.8 | 78.1 | 77807.8 | 226 | 0.01146 | 125.5 | 1.41 |
| CTOX056V1Inj1 | 282.7 | 57.0 | 25506.9 | 177 | 0.00790 | 67.7 | 0.85 |
| CTOX056V1Inj2 | 1116.1 | 58.9 | 77926.8 | 223 | 0.01337 | 186.6 | 1.59 |
| CTOX056V2 | 652.2 | 64.7 | 55623.6 | 226 | 0.01011 | 113.4 | 1.34 |
| CTOX057V2Inj1 | 1288.2 | 124.7 | 97836.3 | 175 | 0.01100 | 202.9 | 1.27 |
| CTOX057V2Inj2 | 893.0 | 160.4 | 68015.1 | 175 | 0.00706 | 118.9 | 0.60 |
| CTOX058V1Inj1 | 1793.9 | 309.2 | 105199.2 | 153 | 0.00903 | 159.8 | 2.05 |
| CTOX058V1Inj2 | 1094.1 | 103.6 | 62673.0 | 201 | 0.00880 | 79.8 | 1.50 |
| CTOX060V1 | 908.1 | 91.4 | 96016.0 | 307 | 0.00609 | 103.6 | 1.39 |
| CTOX061V1 | 392.9 | 66.1 | 47349.9 | 281 | 0.00607 | 82.6 | 1.66 |
| CTOX061V2 | 428.6 | 56.8 | 57369.2 | 314 | 0.00566 | 86.8 | 1.19 |
| CTOX062V1 | 1048.4 | 58.5 | 79078.3 | 196 | 0.01413 | 217.5 | 1.19 |
| CTOX062V2Inj1 | 622.3 | 59.5 | 55915.5 | 282 | 0.00758 | 103.0 | 1.11 |
| CTOX062V2Inj2 | 1280.0 | 67.5 | 97650.3 | 200 | 0.01478 | 277.5 | 1.94 |
| CTOX063V1 | 162.4 | 52.1 | 21879.4 | 249 | 0.00277 | 25.4 | 0.67 |
| CTOX063V2 | 2093.8 | 82.8 | 173650.3 | 227 | 0.01399 | 339.5 | 3.84 |
| CTOX064V1 | 1177.2 | 62.6 | 140400.7 | 312 | 0.00904 | 209.7 | 2.82 |
| CTOX064V2 | 649.5 | 58.0 | 66641.2 | 307 | 0.00764 | 107.3 | 1.74 |
| CTOX065V1 | 1158.9 | 52.0 | 102597.7 | 252 | 0.01198 | 287.6 | 4.56 |
| CTOX065V2 | 282.5 | 47.3 | 34469.9 | 253 | 0.00594 | 69.2 | 1.18 |
| CTOX066V1 | 1406.5 | 84.4 | 107952.8 | 204 | 0.01447 | 228.7 | 1.73 |
| CTOX067V1 | 574.0 | 86.2 | 65329.8 | 198 | 0.00771 | 144.3 | 1.39 |
| CTOX067V2 | 426.0 | 57.4 | 58841.2 | 309 | 0.00552 | 74.2 | 0.84 |
| CTOX068V1 | 4207.0 | 90.5 | 298968.9 | 249 | 0.01484 | 613.3 | 4.85 |
| CTOX068V2Inj1 | 2173.4 | 233.2 | 178169.1 | 197 | 0.00947 | 100.2 | 1.02 |
| CTOX068V2Inj2 | 3235.2 | 385.3 | 274613.4 | 201 | 0.00934 | 401.6 | 3.04 |
| CTOX069V1 | 994.6 | 221.8 | 102194.6 | 198 | 0.00532 | 34.6 | 0.34 |
| CTOX071V1 | 731.2 | 73.3 | 78617.4 | 257 | 0.00939 | 164.7 | 1.82 |
| CTOX071V2 | 685.3 | 75.8 | 75959.5 | 221 | 0.00919 | 180.5 | 2.23 |
| CTOX072V1 | 941.8 | 70.9 | 61704.9 | 225 | 0.01102 | 116.9 | 1.14 |
| CTOX072V2Inj1 | 1627.7 | 65.7 | 114453.1 | 227 | 0.01344 | 210.4 | 1.77 |
| CTOX072V2Inj2 | 1242.5 | 71.4 | 96650.4 | 256 | 0.01069 | 154.6 | 1.28 |
| CTOX074V1 | 5032.4 | 261.2 | 279267.4 | 223 | 0.01172 | 464.8 | 2.67 |
| CTOX074V2Inj1 | 911.9 | 67.5 | 86515.1 | 281 | 0.00813 | 100.2 | 1.11 |
| CTOX074V2Inj2 | 2049.5 | 95.4 | 176294.3 | 278 | 0.01143 | 268.3 | 2.46 |
| CTOX075V1 | 2461.3 | 126.5 | 177629.0 | 224 | 0.01364 | 206.8 | 0.61 |
| CTOX078V1 | 2519.6 | 80.7 | 175846.1 | 199 | 0.01762 | 444.7 | 2.32 |
| CTOX079V1 | 556.0 | 77.7 | 56845.2 | 227 | 0.00854 | 160.3 | 1.30 |
| CTOX079V2 | 862.1 | 62.4 | 80591.2 | 275 | 0.00942 | 154.8 | 1.55 |
